# REST contributes to AKI-to-CKD transition through inducing ferroptosis in renal tubular epithelial cells

**DOI:** 10.1172/jci.insight.166001

**Published:** 2023-06-08

**Authors:** Shuiqin Gong, Aihong Zhang, Mengying Yao, Wang Xin, Xu Guan, Shaozong Qin, Yong Liu, Jiachuan Xiong, Ke Yang, Li Xiong, Ting He, Yinghui Huang, Jinghong Zhao

**Affiliations:** Department of Nephrology, The Key Laboratory for the Prevention and Treatment of Chronic Kidney Disease of Chongqing, Chongqing Clinical Research Center of Kidney and Urology Diseases, Xinqiao Hospital, Army Medical University (Third Military Medical University), Chongqing, China.

**Keywords:** Nephrology, Fibrosis, Hypoxia

## Abstract

Ischemic-reperfusion injury (IRI) is a major pathogenic factor in acute kidney injury (AKI), which directly leads to the hypoxic injury of renal tubular epithelial cells (RTECs). Although emerging studies suggest repressor element 1–silencing transcription factor (REST) as a master regulator of gene repression under hypoxia, its role in AKI remains elusive. Here, we found that REST was upregulated in AKI patients, mice, and RTECs, which was positively associated with the degree of kidney injury, while renal tubule–specific knockout of *Rest* significantly alleviated AKI and its progression to chronic kidney disease (CKD). Subsequent mechanistic studies indicated that suppression of ferroptosis was responsible for REST-knockdown-induced amelioration of hypoxia-reoxygenation injury, during which process Cre-expressing adenovirus–mediated REST downregulation attenuated ferroptosis through upregulating glutamate-cysteine ligase modifier subunit (GCLM) in primary RTECs. Further, REST transcriptionally repressed GCLM expression via directly binding to its promoter region. In conclusion, our findings revealed the involvement of REST, a hypoxia regulatory factor, in AKI-to-CKD transition and identified the ferroptosis-inducing effect of REST, which may serve as a promising therapeutic target for ameliorating AKI and its progression to CKD.

## Graphical abstract

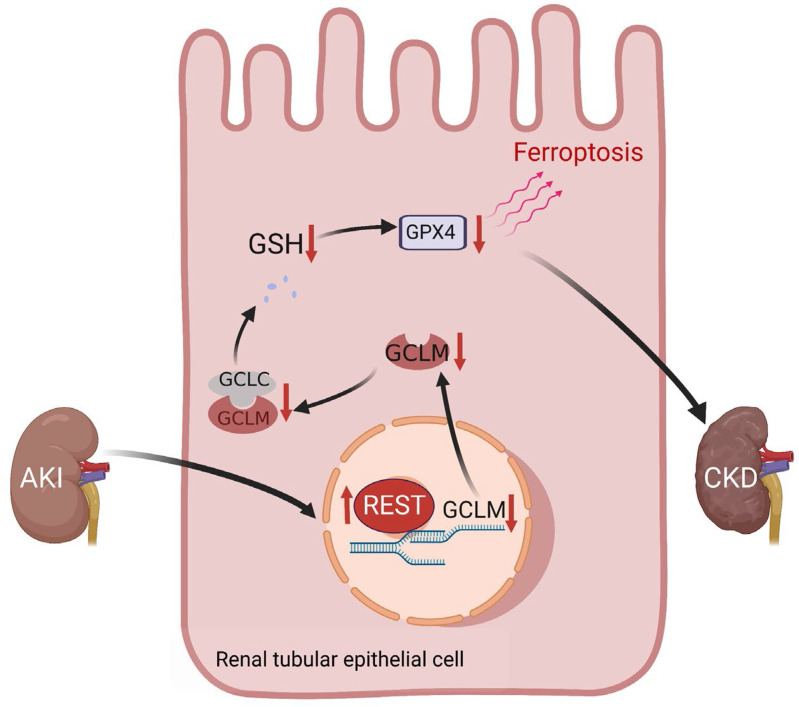

## Introduction

Acute kidney injury (AKI), a severe clinical syndrome characterized by a rapid and sharp decline in renal function, manifests as proximal tubule damage and increased levels of serum creatinine (Scr) and blood urea nitrogen (BUN), and has high morbidity and mortality (1, 2). Worse still, a substantial proportion of patients with AKI gradually progresses to chronic kidney disease (CKD) (3). According to one statistical analysis, AKI patients have a 2.67-fold and 4.81-fold increased risk of CKD and end-stage renal disease, respectively (4). However, there is still a lack of efficient drug-based therapies to cure AKI and/or retard its transition to CKD. Moreover, the precise molecular mechanisms of AKI have not been completely clarified, which impedes the development of effective clinical treatments (5).

Accumulated evidence suggests renal tubular injury as the typical characteristic of AKI, as well as the main initiator of AKI-to-CKD transition (6), since the maladaptive repair of damaged renal tubular epithelial cells (RTECs) under AKI conditions leads to abnormal renal structure and function (7). Intensive research, including ours, has shed light on the underlying mechanisms, including hypoxia, cell cycle arrest, sustained oxidative stress, and mitochondrial dysfunction (8). Further studies reveal that RTEC death contributes to AKI, while protecting these RTECs from death can alleviate AKI (9). Recent findings demonstrated ferroptosis to be involved both in the pathogenesis of AKI and its progression to CKD (10). Ferroptosis is a type of iron-dependent regulated cell death that manifests as overaccumulation of lipid reactive oxygen species (ROS), disruption of polyunsaturated fatty acid metabolism, dysregulation of ferroptosis-suppressing systems, and mitochondrial injury (11–13). Although small-molecule ferroptosis inhibitors, such as liproxstatin-1 and ferrostatin-1 (Fer-1), have been shown to protect against AKI (14, 15), the detailed pathogenic mechanisms of ferroptosis in the transition of AKI to CKD remain elusive.

Repressor element 1–silencing transcription factor (REST) belongs to one of the largest classes of transcription factors with a C2H2- or Krüppel-type zinc finger in humans (16). REST can bind to the promoter region of its target genes, thereby inhibiting their transcription by regulating chromatin structure or suppressing the basal transcriptional machinery (17). REST is a master regulator of gene repression in hypoxia and represses 20% of hypoxia-related genes (18). Of note, acute tubular hypoxia is considered a typical characteristic of ischemia-reperfusion injury–induced (IRI-induced) AKI (19), indicating that REST may play an indispensable role in AKI. Although emerging studies have reported that REST participates in the pathological process of cerebral IRI (20–22), its role and mechanism in IRI-induced AKI remain unclear.

In the present study, we revealed that the renal tubule–specific knockout of *Rest* can alleviate IRI-induced AKI and attenuate AKI-to-CKD transition by inhibiting ferroptosis through transcriptional upregulation of glutamate-cysteine ligase modifier subunit (GCLM) via direct binding to its promoter region. Therefore, targeted inhibition therapy of REST-mediated ferroptosis may serve as a novel therapeutic strategy for ameliorating AKI and its transition to CKD.

## Results

### REST is induced in AKI models in vitro and in vivo.

**Figure 1:**
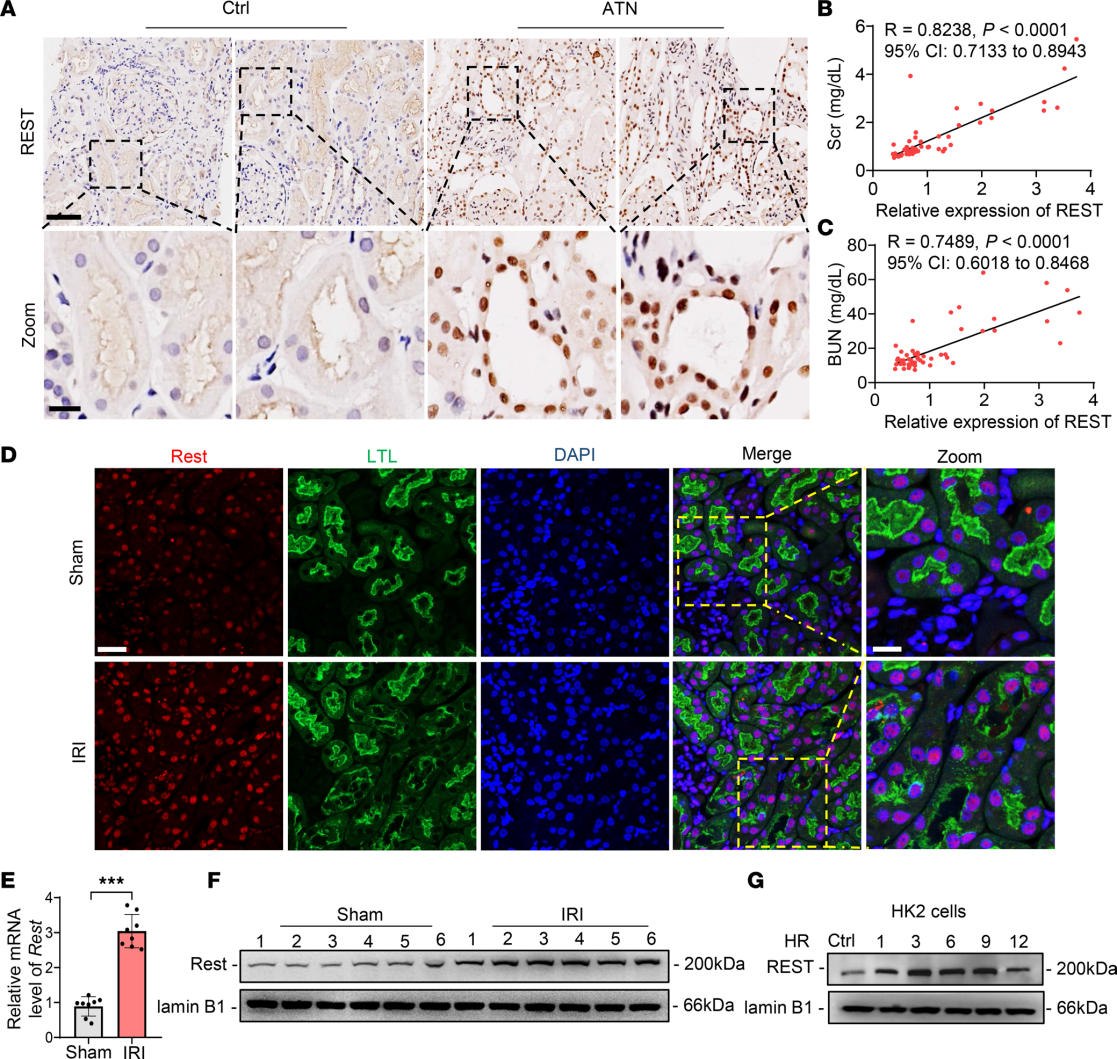
REST is upregulated in AKI both in vitro and in vivo. (**A**) Representative immunohistochemical staining of REST in ATN patients (*n* = 20) or control (*n* = 34). Scale bar: 100 μm (top); 25 μm (down). (**B** and **C**) Correlation analysis between REST and Scr (**B**) or BUN (**C**). (**D**–**F**) Immunofluorescence (**D**), qPCR (**E**), and Western blot (**F**) analyses of REST in kidney tissues of sham and IRI-induced AKI mice. Scale bar: 50 μm (left); 25 μm (right). LTL, *Lotus* *tetragonolobus* lectin. (**G**) Western blot analysis of REST in HK2 cells under HR condition (*n* = 3). Data are shown as mean ± SD and were analyzed by Pearson’s correlation analysis (**B** and **C**) or 2-tailed, unpaired Student’s *t* test (**E**). ***P < 0.001.

We first assessed the expression of REST in different organs and found moderate expression in the heart, liver, spleen, lung, and kidney, as well as lower levels in the brain (Supplemental Figure 1, A and B; supplemental material available online with this article; https://doi.org/10.1172/jci.insight.166001DS1). High-throughput transcriptome data (GEO GSE52004) showed that *Rest* was significantly upregulated in AKI mice (Supplemental Figure 1C). To verify the sequencing data, renal biopsy specimens from 20 patients with acute tubular necrosis (ATN) and 34 patients with no detectable ATN were enrolled in this study (Supplemental Table 1). The immunochemical staining and statistical analysis indicated an upregulated REST expression level in ATN, which was positively correlated with the markers of kidney injury, Scr and BUN (Figure 1, A–C). To further investigate the role of REST in AKI, both IRI- and cisplatin-induced AKI mouse models were established (Supplemental Figure 2, A–F). REST was induced at both transcriptional and translational levels in renal tubules of AKI mice (Figure 1, D–F, and Supplemental Figure 3, A–C) and IRI-induced AKI-to-CKD mice (Supplemental Figure 3D). Further, the protein levels of REST were upregulated under hypoxia-reoxygenation (HR) injury in tubular epithelial cell lines derived from human (HK2), as well as mouse primary RTECs and rat (NRK52E) (Figure 1G and Supplemental Figure 3, E and F). REST expression was also dose-dependently induced by cisplatin (Supplemental Figure 3, G and H). Collectively, these results suggest a transcriptional and translational upregulation of REST in AKI both in vitro and in vivo.

### Renal tubule–specific knockout of Rest alleviated AKI.

**Figure 2:**
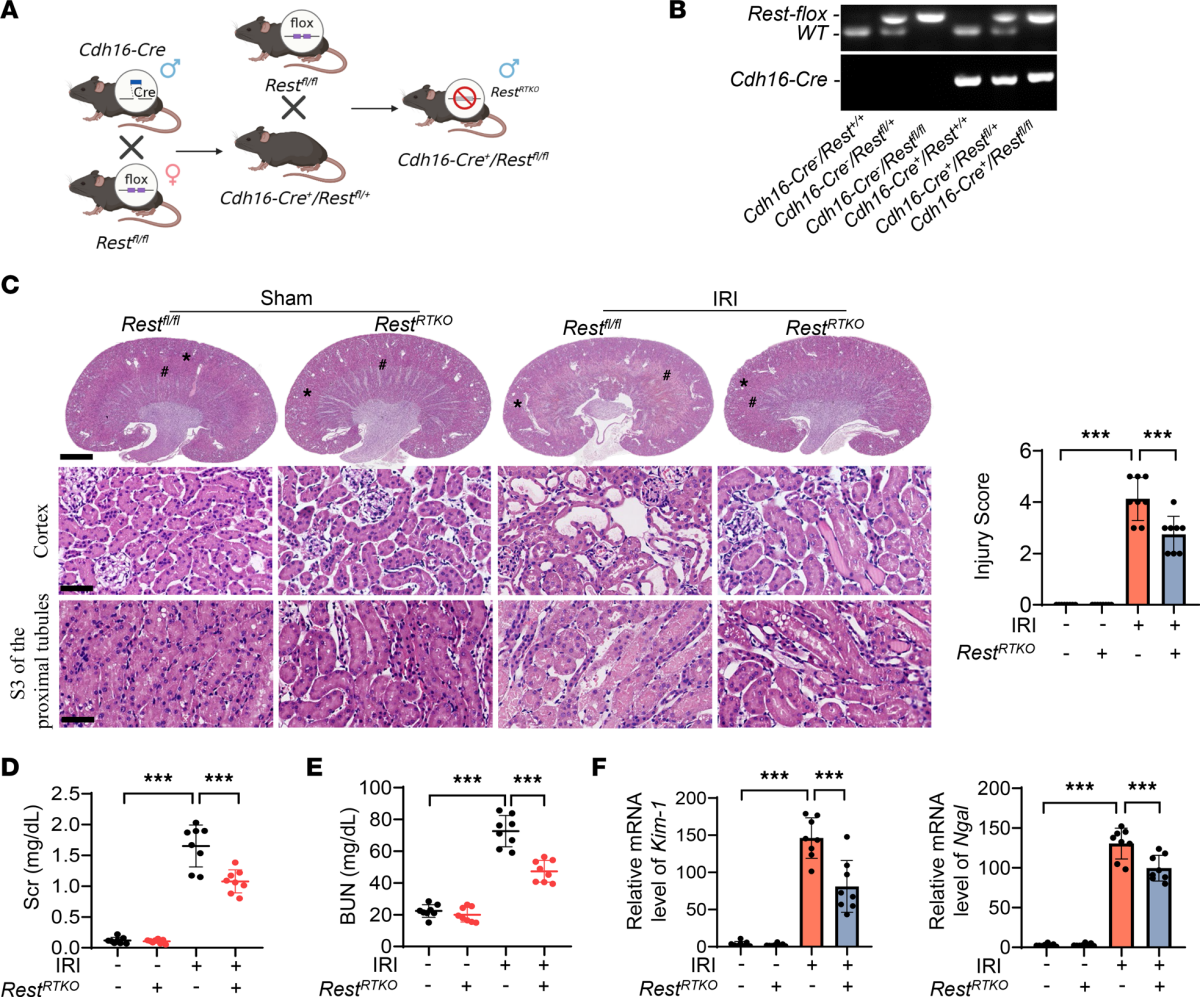
Conditional knockout of *Rest* in renal tubular epithelial cells protects against AKI. (**A** and **B**) Reproductive strategy of renal tubular conditional *Rest*-knockout mice (**A**) and genotyping confirmation of mice at the age of 2 weeks (**B**). (**C**) H&E staining and injury score of the kidneys from *Rest^fl/fl^* and *Rest^RTKO^* mice with or without IRI. *, cortex; #, S3 of the proximal tubules (*n* = 8 mice per group). Scale bars: 1.25 mm (top) and 50 μm (middle and bottom). (**D**–**F**) Levels of Scr (**D**) and BUN (**E**) and qPCR analyses of *Kim-1* and *Ngal* levels (**F**) of the kidneys from mice in **C**. Data are shown as mean ± SD and were analyzed by 1-way ANOVA (**C**–**F**). ***P < 0.001.

To investigate the role of REST in AKI, renal tubule–specific *Rest*-knockout (*Rest^RTKO^*) mice were constructed (Figure 2, A and B, and Supplemental Figure 4). *Rest* was successfully knocked out in *Rest^RTKO^* mice compared with *Rest^fl/fl^* mice (Supplemental Figure 5). Pathological damage to the kidney was alleviated in *Rest^RTKO^* mice (Figure 2C). In addition, the levels of Scr and BUN were significantly decreased (Figure 2, D and E), and the expression levels of kidney injury markers, kidney injury molecule-1 (Kim-1) and neutrophil gelatinase–associated lipocalin (Ngal), were also markedly downregulated (Figure 2F). Thus, knockout of *Rest* in RTECs may protect the kidney from IRI-induced AKI.

### REST knockdown reduced ferroptosis in HR model.

**Figure 3:**
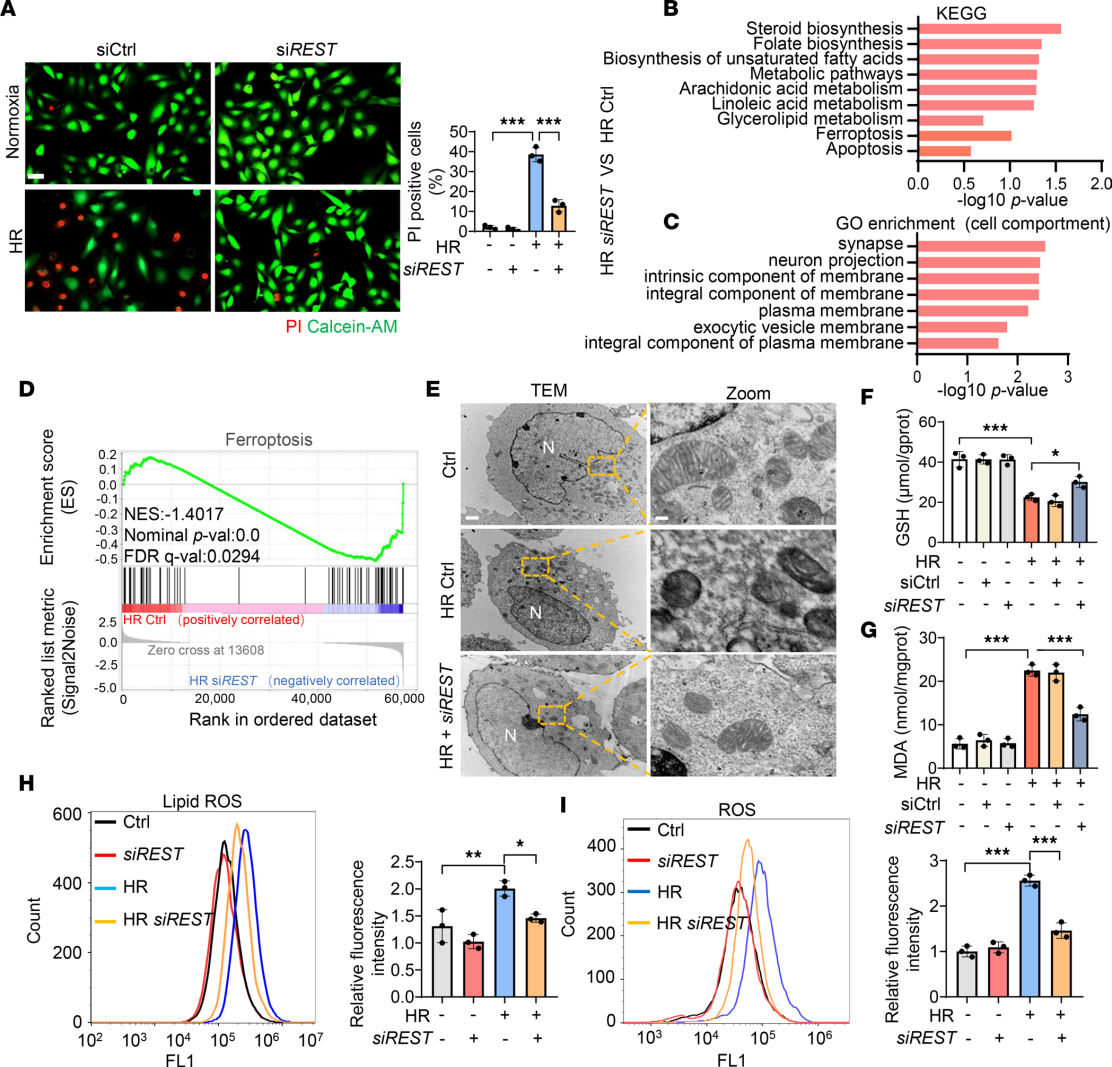
Silencing *REST* relieves ferroptosis under HR condition. HK2 cells were transfected with control or siRNAs against *REST* (*siREST*), and then exposed to HR injury for RNA-seq (*n* = 4). (**A**–**D**) PI/calcein-AM staining (**A**), KEGG pathway classification (**B**), GO enrichment analysis of significant changes in cellular component (**C**), and GSEA enrichment analysis of ferroptosis pathway (**D**) between control and *REST*-knockdown group under HR condition. Scale bar: 50 μm. (**E**–**I**) Representative TEM images of mitochondria (**E**), GSH levels (**F**), MDA levels (**G**), lipid ROS production (**H**), and ROS production (**I**) in different groups (*n* = 3). Scale bar: 1 μm (left), 0.15 μm (right). N, nucleus. Data are shown as mean ± SD and were analyzed by 1-way ANOVA (**A** and **F**–**I**). *P < 0.05; **P < 0.01; ***P < 0.001.

To gain insight into the molecular mechanism underlying the role of REST in AKI, we silenced *REST* in HK2 cells using siRNA (Supplemental Figure 6A). *REST*-knockdown cells showed higher cell viability, fewer PI-positive cells, and lower levels of lactic dehydrogenase (LDH) release compared with the control group under the HR condition (Figure 3A and Supplemental Figure 6, B and C), revealing reduced cell death in the *REST*-knockdown group. Next, we performed whole-transcriptome profiling using RNA sequencing (RNA-seq). Kyoto Encyclopedia of Genes and Genomes (KEGG) analysis showed that ferroptosis was enriched when *REST* was knocked down under the HR condition (Figure 3B). Gene Ontology (GO) analysis revealed strong enrichment of biological membranes (Figure 3C), consistent with the dysregulation of membrane lipid peroxidation that is associated with ferroptosis (23). In addition, we performed gene set enrichment analysis (GSEA), which identified negative enrichment of ferroptosis in the *REST*-knockdown group under the HR condition (Figure 3D).

Consistent with these data, transmission electron microscopy (TEM) observations of HK2 cells following HR injury revealed increased mitochondrial shrinkage, ruptured mitochondrial membranes, and reduced numbers of cristae that were significantly alleviated by knockdown of *REST* (Figure 3E). In addition, *REST* knockdown under HR conditions enhanced the production of glutathione (GSH) and reduced the accumulation of the lipid oxidation product malondialdehyde (MDA), lipid ROS, and ROS (Figure 3, F–I), indicating a reduction in lipid peroxidation. Further, we found that overexpression of *REST* in the HR model increased cell death, which was significantly rescued by the ferroptosis inhibitors, the lipophilic radical scavenger (Fer-1) and iron chelator (deferoxamine, DFO) (11, 24, 25), rather than the apoptosis inhibitor (benzyloxycarbonyl-Val-Ala-Asp [OMe] fluoromethylketone, Z-VAD-FMK), necroptosis inhibitor (necrostatin-1, Nec-1), or autophagy inhibitor (3-methyladenine, 3-MA) (Supplemental Figure 6D).

We also used 2 kinds of ferroptosis inducer (Erastin and RSL3) (26) to investigate whether REST deficiency can alleviate ferroptosis. Interestingly, knockdown of *REST* significantly attenuated cell death induced by RSL3, while it showed no obvious improvement in Erastin-induced ferroptosis (Supplemental Figure 6, E–G). Further, knockdown of *REST* also effectively ameliorated ferroptosis induced by RSL3 in a standard ferroptosis cell line (HT1080 cells) (Supplemental Figure 6, H–L). These observations indicate that knockdown of *REST* can suppress ferroptosis that is mediated by the inhibition of GSH peroxidase 4 (GPX4) rather than cystine deprivation.

To investigate whether REST deficiency can overcome *GPX4* deficiency–induced ferroptosis, we used a cell line with doxycycline-inducible (Dox)-inducible knockdown of *GPX4* (iGPX4KD cells) (27) (Supplemental Figure 7A). As expected, incubation with 1 μM Dox induced significant cell death in iGPX4KD cells, which was rescued by coincubation with Fer-1 or knockdown of *REST* (Supplemental Figure 7, B and C). Lipid peroxidation indices also revealed that both Fer-1 and knockdown of *REST* mitigated ferroptosis in iGPX4KD cells (Supplemental Figure 7, D–F). In addition, knockdown of *REST* and coincubation with Fer-1 further attenuated ferroptosis in iGPX4KD cells (Supplemental Figure 7). These findings suggest that REST deficiency can overcome the dependence of lipophilic radical–trapping agents in iGPX4KD cells. Of interest, knockdown of *REST* had additional effects on the ferroptosis inhibitors (Fer-1 and DFO), as evidenced by restored levels of GSH, MDA, and lipid ROS and improved mitochondrial morphology (Supplemental Figure 8, A–C). These bioinformatic analyses and experimental findings collectively reveal that silencing of *REST* mitigates ferroptosis under HR conditions.

### REST knockdown alleviated ferroptosis through inducing GCLM expression in the HR model.

**Figure 4:**
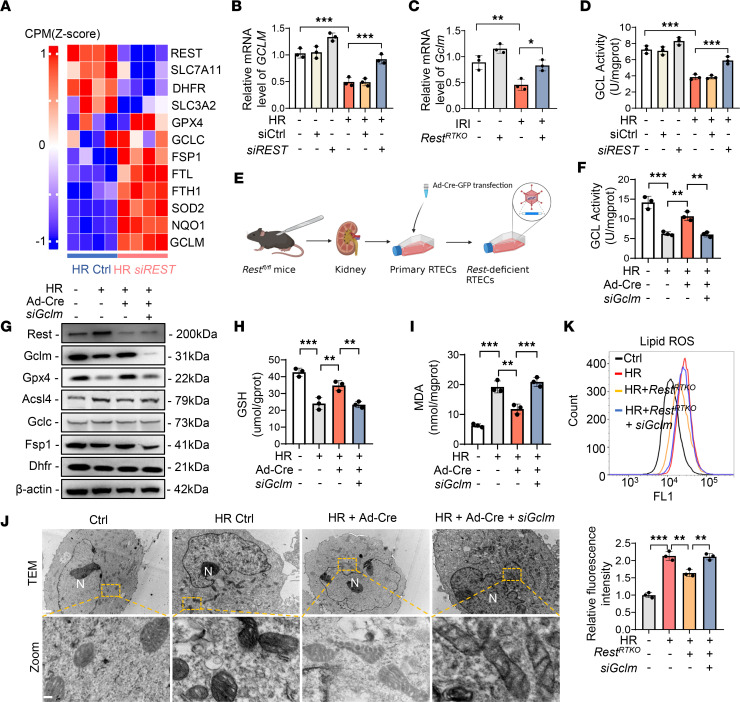
The knockdown of *REST* inhibits ferroptosis via upregulating GCLM expression under HR condition. (**A**) Heatmap analysis of REST and ferroptosis-related genes between control and *REST*-knockdown group under HR condition. (**B** and **C**) qPCR analyses of *GCLM* expression in HK2 cells under normal conditions or HR injury transfected with control or *siREST* (**B**) and renal tubules from *Rest^fl/fl^* or *Rest^RTKO^* mice (**C**). (**D**) GCL activity of cells in **B** (*n* = 3). (**E**–**J**) Primary RTECs from *Rest^fl/fl^* mice were infected with control or Ad-Cre-GFP and cotransfected with control or siRNAs against *GCLM* (*siGCLM*) (**E**), and then subjected to HR injury for analysis of GCL activity (**F**), Western blot analyses of ferroptosis-related proteins REST, GCLM, GPX4, ACSL4, GCLC, FSP1, and DHFR (**G**), GSH levels (**H**), MDA levels (**I**), and TEM observation of ferroptosis (**J**) (*n* = 3). Scale bar: 1 μm (top); 0.15 μm (bottom). N, nucleus. (**K**) Primary RTECs were isolated from *Rest^fl/fl^* and *Rest^RTKO^* mice, and then cotransfected with control or siRNAs against *GCLM* under normal or HR conditions to detect lipid ROS production (*n* = 3). Data are shown as mean ± SD and were analyzed by 1-way ANOVA (**B**–**D**, **F**, **H**, **I**, and **K**). *P < 0.05; **P < 0.01; ***P < 0.001.

To decipher the underlying molecular mechanisms, we first analyzed ferroptosis-related genes by RNA-seq, which showed significant amelioration in the *REST*-knockdown group under the HR condition (Figure 4A). Although the top 10 upregulated genes (transcripts per million > 10) were evaluated in HK2 cells and renal tubules from *Rest^RTKO^* mice (Supplemental Figures 9 and 10), only *GCLM* showed a significant statistical change in both comparisons (Figure 4, B and C), indicating that knockdown of *REST* may promote GCLM expression in both humans and mice.

GCLM is the modifier subunit of glutamate-cysteine ligase (GCL), a rate-limiting enzyme of GSH synthesis (28). Accordingly, rating-limiting enzymes in GSH synthesis were detected (28), but GCL catalytic subunit (GCLC) and GSH synthetase (GSS) showed no significant changes after the silencing of *REST* (Supplemental Figure 11, A and B). Previous studies reported that loss of GCLM only results in decreased activity of GCL and reduced GSH synthesis (29, 30). GCL activity and the expression levels of ferroptosis markers, GPX4 and acyl-CoA synthetase long-chain family member 4 (ACSL4), were remarkably improved in the *REST*-knockdown cells under the HR condition, rather than other ferroptosis-associated proteins (GCLC, dihydrofolate reductase [DHFR], and ferroptosis suppressor protein 1 [FSP1]), apoptosis-associated proteins (B cell leukemia/lymphoma 2 [Bcl-2] and Bcl-2–associated X [Bax]), autophagy-associated proteins (microtubule-associated protein 1A/1B-light chain 3 [LC3] and sequestosome 1 [p62]), or pyroptosis-associated proteins (caspase-1 and GSDMD) (Figure 4D and Supplemental Figure 11, C and D).

To further demonstrate whether the regulation of GCLM by REST is involved in ferroptosis, primary RTECs from *Rest^fl/fl^* mice were isolated and infected with Cre recombinase adenovirus (Ad-Cre) to knock out *Rest* (Figure 4E and Supplemental Figure 12A). The GCL activity, GSH level, MDA level, the expression of GPX4 and ACSL4, and TEM observation again strongly supported the notion that a reduction in GCLM following *Rest* knockout enhanced ferroptosis under the HR condition (Figure 4, F–J, and Supplemental Figure 12, B–E). Primary *Rest-*deficient RTECs were also isolated and cultured from *Rest^RTKO^* mice for lipid ROS detection, which showed a significant aggravation when *Gclm* was knocked down (Figure 4K and Supplemental Figure 12F). These results suggest that *Rest* knockout inhibited ferroptosis by increasing GCLM expression.

### REST transcriptionally represses GCLM via directly binding to its promoter region.

**Figure 5:**
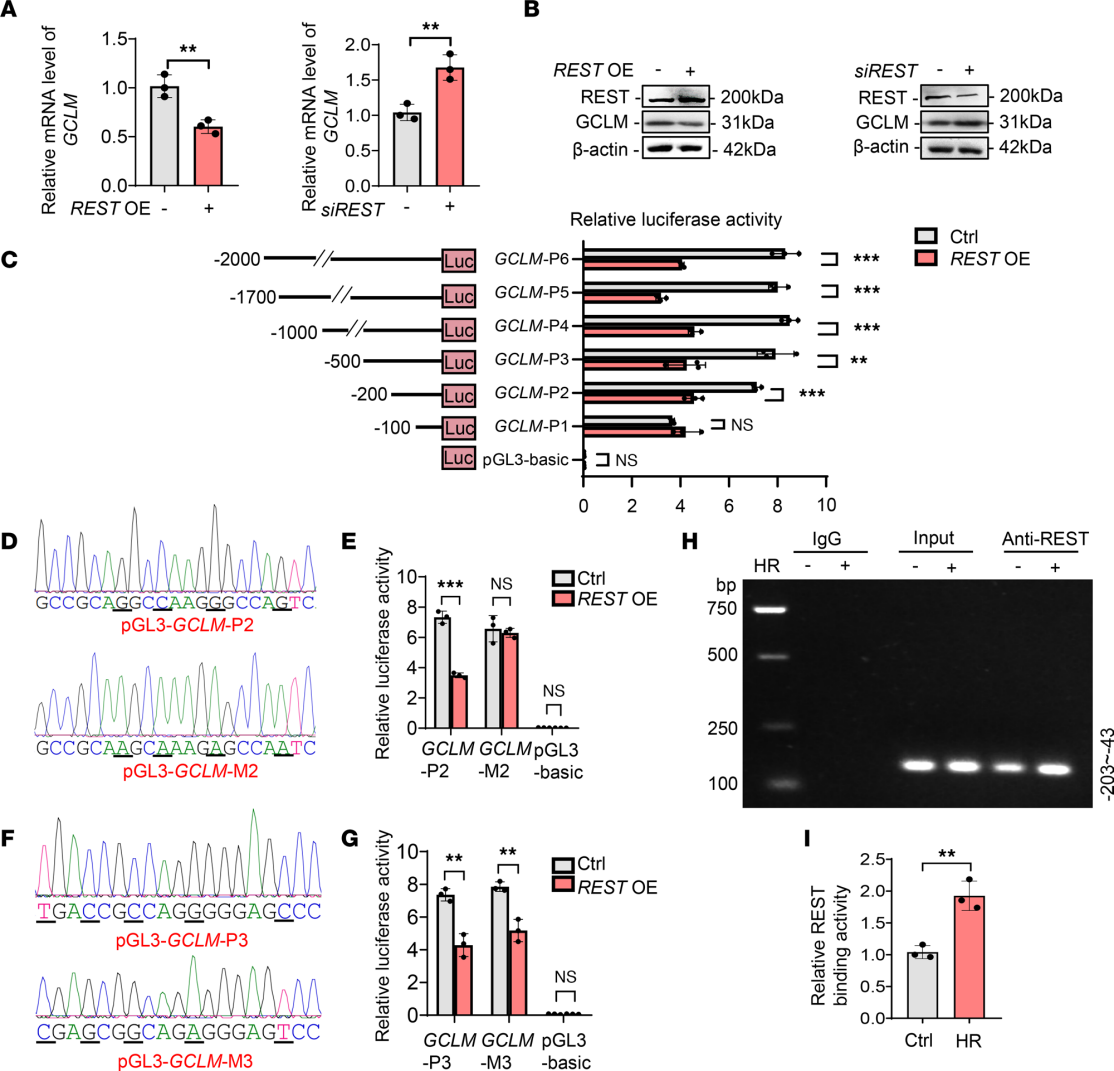
REST suppresses GCLM transcription through directly binding to its promoter region. (**A** and **B**) qPCR (**A**) and Western blot (**B**) analyses of GCLM in HK2 cells transfected with control or *siREST*, or *REST* overexpression plasmids (*REST* OE) (*n* = 3). (**C**) HK2 cells were cotransfected with pRL-TK plasmids and pGL3-basic or recombinant reporter plasmids containing various fragments of the *GCLM* promoter region with or without *REST* overexpression, after which cells were harvested for luciferase assay (*n* = 3). (**D**) The pGL3-*GCLM*-P2 and pGL3-*GCLM*-M2 fragments (mutant bases are underlined in the sequencing results). (**E**) HK2 cells were cotransfected with pRL-TK and pGL3-*GCLM*-P2 or pGL3-*GCLM*-M2 with or without *REST* overexpression, and harvested for luciferase assay (*n* = 3). (**F**) The pGL3-*GCLM*-P3 and pGL3-*GCLM*-M3 fragments (mutant bases are underlined in the sequencing results). (**G**) HK2 cells were cotransfected with pRL-TK and pGL3-*GCLM*-P3 or pGL3-*GCLM*-M3 with or without *REST* overexpression, and harvested for luciferase assay (*n* = 3). (**H** and **I**) Cells were exposed to control or HR injury and collected for ChIP assay. REST antibody was immunoprecipitated with DNA fragments. The precipitated DNA was amplified using PCR primers covering the *GCLM* promoter region (–43 to –203). The PCR and qPCR analyses are respectively shown in **H** and **I** (*n* = 3). Data are shown as mean ± SD and were analyzed by 2-tailed, unpaired Student’s *t* test (**A**, **C**, **E**, **G**, and **I**). **P < 0.01; ***P < 0.001. NS, no significance.

To further investigate the relationship between REST and GCLM, the mRNA and protein levels of GCLM under different conditions were first compared (Figure 5, A and B). The results showed a strong negative correlation between GCLM and REST. We next searched for potential binding sites of REST in *GCLM* using JASPR, which predicted 4 sites.

To explore this deduction, 6 truncated plasmids comprising various lengths of nucleotide sequences were designed using *GCLM*’s promoter sequence. Next, these plasmids were individually transfected into HK2 cells. Cells transfected with *REST* overexpression plasmids significantly decreased the luciferase activities of reporter plasmids pGL3-*GCLM*-P2 to pGL3-*GCLM*-P6, but no statistical difference was observed in the pGL3-*GCLM*-P1 group (Figure 5C), suggesting that the binding site is contained within the sequence –200 to –100 nucleotides upstream of the *GCLM* transcription start site. According to our bioinformatics analysis, the portion containing nucleotides –152 to –132 (GCCGCAGGCCAAGGGCCAGTC) should contain the binding site. We then mutated the sequence to GCCGCAAGCAAAGAGCCAATC (Figure 5D), which nearly abolished the observed difference in luciferase activity (Figure 5E). However, mutation of another predicted binding site in pGL3-*GCLM*-P3 did not result in distinct changes (Figure 5, F and G), further supporting our analysis. Chromatin immunoprecipitation (ChIP) also verified direct binding of REST to the *GCLM* promoter region (–203 to –43), which could be enhanced by HR (Figure 5, H and I). These findings reveal that REST directly binds to the promoter region of *GCLM* to inhibit its expression.

### Conditional knockout of Rest in RTECs ameliorates IRI-induced ferroptosis and AKI-to-CKD transition.

**Figure 6:**
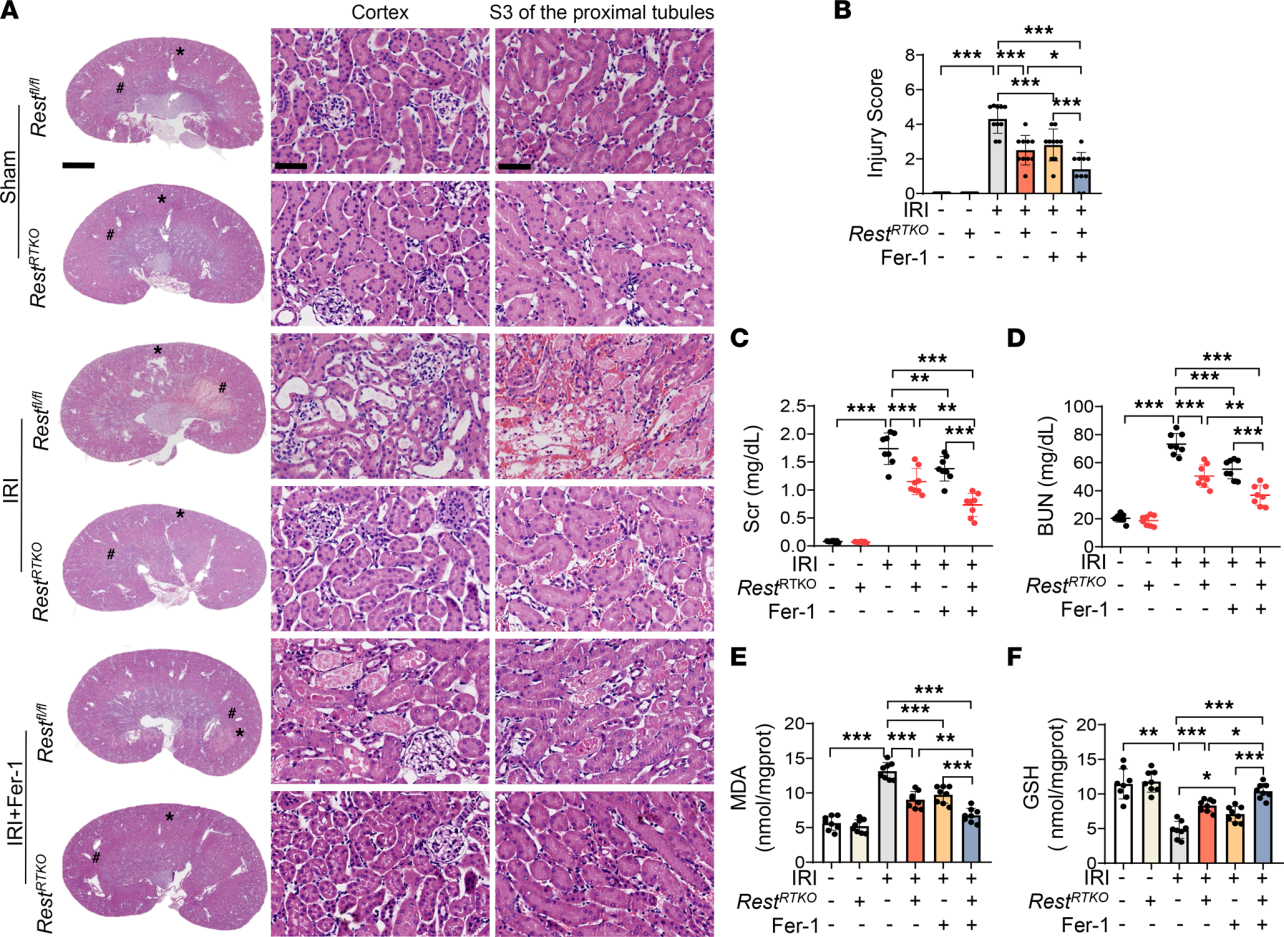
Renal tubule–specific knockout of *Rest* alleviates lipid peroxidation in IRI-induced AKI mice. *Rest^RTKO^* and *Rest^fl/fl^* mice were subjected to IRI and sacrificed on reperfusion day 1. Ferrostatin-1 (Fer-1; 5 mg/kg) or normal saline was intraperitoneally injected into the mice at the onset of reperfusion. The following indices were evaluated (*n* = 8 mice per group). (**A** and **B**) H&E staining (**A**) and injury scores (**B**) of the kidneys from all groups. *, cortex; #, S3 of the proximal tubules. Scale bars: 1.25 mm (left) and 50 μm (middle and right). (**C** and **D**) Levels of Scr (**C**) and BUN (**D**) from mice in **A**. (**E** and **F**) GSH levels (**E**) and MDA levels (**F**) of the kidneys from mice in **A**. Data are shown as mean ± SD and were analyzed by 1-way ANOVA (**B**–**F**). *P < 0.05; **P < 0.01; ***P < 0.001.

To further explore the role of REST in vivo, *Rest^RTKO^* mice were subjected to IRI, which exhibited significant amelioration of renal injury, as evidenced by ameliorated pathological damage and reduced Scr and BUN levels (Figure 6, A–D). Further, the upregulated MDA and downregulated GSH in *Rest^fl/fl^* AKI mice were also restored in *Rest^RTKO^* mice (Figure 6, E and F), suggesting an alleviated lipid peroxidation. Consistent with the in vitro assays (Supplemental Figure 8, A–C), intraperitoneally injected Fer-1 alleviated renal injury and the disturbed levels of MDA and GSH in *Rest^fl/fl^* mice, which showed a further improvement in *Rest^RTKO^* mice (Figure 6, A–F), suggesting an additional effect of Fer-1 and REST deficiency on renal protection.

**Figure 7:**
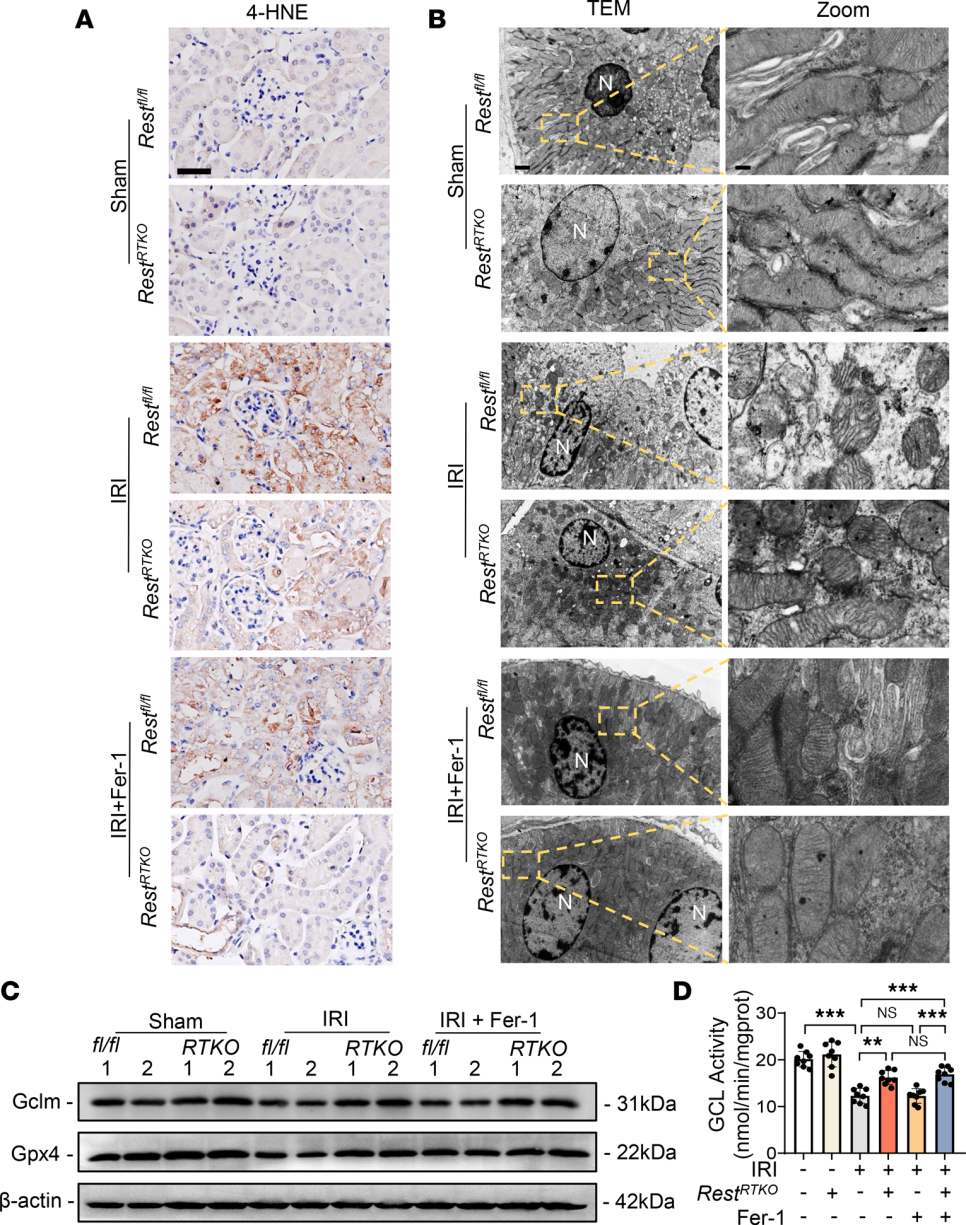
Renal tubule–specific knockout of *Rest* alleviates AKI through upregulating GCLM expression to inhibit ferroptosis. *Rest^RTKO^* mice and *Rest^fl/fl^* mice were subjected to IRI and sacrificed on reperfusion day 1. Ferrostatin-1 (Fer-1; 5 mg/kg) or normal saline was intraperitoneally injected into the mice at the onset of reperfusion. The following indices were evaluated (*n* = 8 mice per group). (**A**) Representative immunohistochemical staining of 4-HNE from all groups. Scale bar: 50 μm. (**B**–**D**) TEM observation of mitochondria (**B**), representative Western blot analyses of GCLM and GPX4 (**C**), and GCL activity (**D**) of the kidneys from all groups. Scale bar: 1 μm (left); 0.15 μm (right). N, nucleus. Data are shown as mean ± SD and were analyzed by 1-way ANOVA (**D**). **P < 0.01; ***P < 0.001. NS, no significance.

As anticipated, the disordered ferroptosis markers in the AKI mouse model, including increased 4-hydroxynonenal (4-HNE), damaged mitochondrial morphology, and dysregulated expression levels of GPX4, were also ameliorated in *Rest^RTKO^* mice, with a further amelioration in Fer-1–treated mice (Figure 7, A–C). However, only REST deficiency, rather than Fer-1, restored the downregulated GCLM expression and reduced GCL activity in AKI mice (Figure 7, C and D), indicating a unique role of REST and the different mechanisms of ameliorating ferroptosis between REST deficiency and Fer-1.

**Figure 8:**
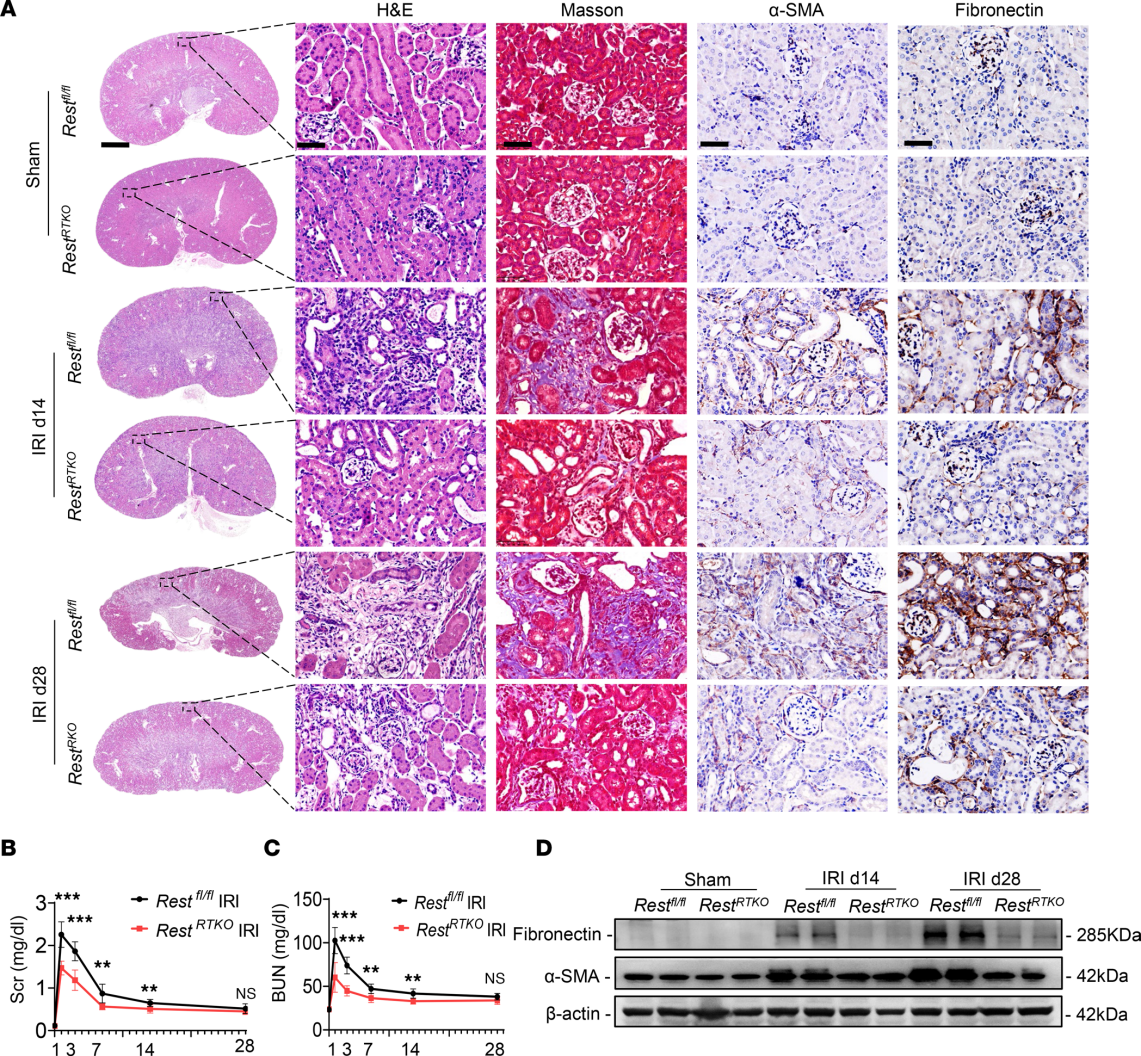
Renal tubule–specific *Rest* knockout retards the transition from AKI to CKD. (**A**) *Rest^fl/fl^* and *Rest^RTKO^* mice were subjected to IRI or sham, and then sacrificed on the 14th and 28th day after reperfusion. The kidneys were removed for H&E and Masson’s staining, and immunohistochemical staining of α-SMA and fibronectin. Scale bars: 1.25 mm (far left) and 50 μm (others) (*n* = 8 mice per group). (**B** and **C**) Levels of Scr (**B**) and BUN (**C**) in mice in **A**. (**D**) Western blot analysis of the protein levels of fibronectin and α-SMA in the renal tubules from mice in **A**. Data are shown as mean ± SD and were analyzed by 2-tailed, unpaired Student’s *t* test (**B** and **C**). **P < 0.01, ***P < 0.001. NS, no significance.

**Figure 9:**
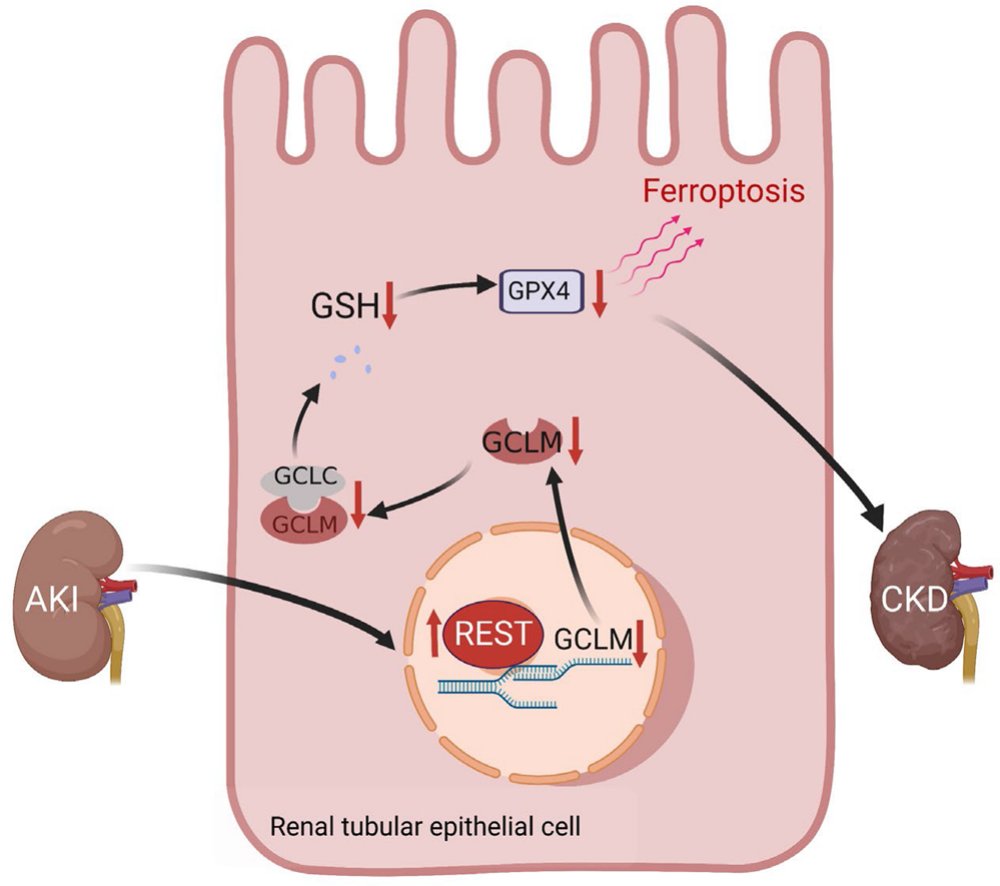
Proposed model for the role and mechanism of REST in suppressing ferroptosis and alleviating the transition from AKI to CKD.

To further explore whether *REST* knockout affects the transition of AKI to CKD, we established IRI-induced AKI-to-CKD models. As anticipated, *Rest^RTKO^* mice exhibited obvious attenuation of renal fibrosis, as evaluated by hematoxylin and eosin (H&E) staining, Masson’s staining, and immunohistochemical staining of α-SMA and fibronectin (Figure 8A), along with reduced Scr and BUN levels (Figure 8, B and C). Moreover, the expression of α-SMA and fibronectin was significantly rescued in *Rest^RTKO^* mice (Figure 8D). In addition, we detected the expression of GCLM and GPX4 in IRI-induced AKI to CKD. *Rest^RTKO^* mice subjected to IRI exhibited significantly enhanced GCLM expression, and rescued the expression of GPX4 on reperfusion day 14 and day 28 (Supplemental Figure 13). These findings support the notion that conditional knockout of *Rest* in RTECs reduced kidney injury and renal fibrosis in an IRI-induced AKI-to-CKD model (Figure 9).

## Discussion

AKI, a clinical syndrome with high incidence and mortality, often progresses to CKD with malpractice. As the pathogenesis of AKI remains unknown, targeted therapy is lacking clinically, with the exception of hemodialysis. In our investigation, a series of in vitro and in vivo experiments were conducted to confirm that *Rest^RTKO^*-mediated alleviation of the AKI-to-CKD transition could be attributed to the REST/GCLM pathway. REST directly bound to the promoter region of *GCLM* (–152 to –132, GCCGCAGGCCAAGGGCCAGTC) to repress its transcription, while knockdown of *REST* increased GCLM transcription and enhanced GCL enzymatic activity, leading to upregulation of GSH and GPX4 to reduce lipid peroxidation and ferroptosis in AKI. Therefore, REST may serve as a potential target for AKI-to-CKD therapy.

As one of the largest classes of transcription factors in humans, REST participates in neuronal differentiation, axon growth, vesicle transport, and ionic conductance through regulating its downstream genes (31). Recently, universal molecular functions of REST have been found with cellular and tissue specificity (32). REST is expressed in the nucleus when suffering an IRI in the brain, leading to neuronal injury (21). When the expression of REST was suppressed, inflammation and oxidative stress improved in the cerebrum (20). Of note, several REST inhibitors, including valproic acid and X5050, have been shown to be clinically effective in rescuing many brain diseases such as seizures (31, 33). Meanwhile, in the aging kidney, REST expression was enhanced in podocytes, while conditional knockout of *REST* in podocytes induced cell apoptosis (34). However, no previous studies to our knowledge evaluated the role and mechanism of REST in AKI.

In our study, RNA-seq of RTECs with *REST* knockdown under HR, primary RTECs of mice with *Rest* knockout, histological examination of *Rest^RTKO^* mouse kidneys, and TEM observations collectively demonstrated that ferroptosis was one of the key regulatory pathways through which REST contributed to AKI-to-CKD transition. Further, the ferroptosis inhibitors significantly restored cell viability upon overexpression of *REST* in the HR model, rather than the inhibitors of apoptosis, autophagy, or necrosis. These findings suggest that REST mainly induces ferroptosis, instead of other forms of cell death in the HR model. Nevertheless, we cannot exclude the roles of other genes in AKI, since the protection induced by REST deficiency is partial in vivo.

As a recently identified type of programmed cell death, ferroptosis participates in the occurrence and development of AKI (10, 35). Although the use of ferroptosis inhibitors can improve the prognosis of AKI (14, 36), the pathogenesis of ferroptosis in AKI remains unclear. As reported previously, GPX4 is identified as the gatekeeper that limits lipid peroxidation associated with ferroptosis (37). GPX4 can reduce lipid hydroperoxide to nontoxic lipid alcohol in the membrane to inhibit lipid peroxidation, while dysfunction of GPX4 has been shown to hypersensitize mice to tubular necrosis during AKI (38). Accordingly, knockout or inactivation of GPX4 is a classical method to induce ferroptosis (37). Recently, pathways parallel to the GPX4 pathway were discovered in succession, including GCH1/BH4/DHFR and FSP1/CoQ10 (39). FSP1 mainly localizes to lipid droplets and the plasma membrane, where it can directly reduce lipid radicals or indirectly recycle α-tocopherol (40). Meanwhile, GTP cyclohydrolase 1/tetrahydrobiopterin counteracts ferroptosis through lipid remodeling (41), but blockade of DHFR synergizes with GPX4 inhibition to induce ferroptosis (42). In our research, screening of the key suppressors of ferroptosis revealed that GPX4 was upregulated by silencing of *REST*, while DHFR and FSP1 were not. Further, the upregulation of REST in AKI suppressed GCLM transcription and reduced GCL enzymatic activity, leading to the downregulation of GSH and GPX4. Meanwhile, knockdown of *REST* significantly increased GSH synthesis, restored GPX4 expression, and ultimately attenuated ferroptosis, which had an additive effect on the ferroptosis inhibitors Fer-1 and DFO. Of note, they mitigate ferroptosis through different mechanisms, since DFO-mediated cleavage of iron effectively suppresses the Fenton reaction, thereby reducing hydroxyl radicals and lipophilic antioxidants, while Fer-1 acts as a lipophilic radical–trapping agent (39). Thus, inhibition of the REST/GCLM axis is highly expected to be a novel therapeutic target for AKI.

Modified subunit GCLM and catalytic subunit GCLC together constitute GCL. GCL and GSS are 2 key enzymes in GSH synthesis (43). Knockout of *GCLM* results in suppression of GCL activity and further inhibition of GSH synthesis, as has been found in myocardial IRI (44). It was also reported that both HR injury and IRI significantly reduced the expression of GCLM compared with the control group (45, 46). Our research also verified that decreased expression of GCLM was responsible for the decreased activity of GCL, and that GCL activity was enhanced after the resumption of GCLM to promote GSH synthesis, which directly retarded ferroptosis. In HR injury and IRI, elevated REST levels increase its binding to and repression of GCLM, which was confirmed by dual-luciferase reporter assay and ChIP results, indicating further induction of ferroptosis. Further, multiple studies have reported GSH as a vital contributor to preventing lipid peroxidation and protecting cells from ferroptosis (25, 47, 48), indicating the potential effect of enhancing the production of GCLM or GSH in treating AKI.

IRI is the most common pathogenesis that induces AKI in clinics, and generally occurs in the cerebrum, intestines, heart, lungs, and liver, during which process ferroptosis plays a vital role. Inactivation of GPX4 leads to AKI in mice, which can be improved by lipid peroxidation inhibitors (14). Further, ferroptosis inhibitors can alleviate the damage induced by IRI in the intestines (49) and myocardium (50). The above research indicates that hypoxia and ferroptosis are widespread in diseased organs exposed to IRI, and the potential of REST, a hypoxia regulator and ferroptosis inducer, as a therapeutic target in other organs also deserves further investigation.

In conclusion, REST is identified as a significant cause of ferroptosis induced in AKI. REST acts by suppressing GCLM expression, leading to decreased GCL activity and limited GSH synthesis that indicates higher sensitivity to ferroptosis. *Rest^RTKO^* alleviates AKI and retards the progression of AKI to CKD. As the mechanism of REST in AKI has not been mentioned in previous studies, targeted therapy of REST has enormous promise to improve AKI, prevent kidney fibrosis, and contribute to the recovery of renal function.

## Methods

Additional details for methods are provided in the Supplemental Methods.

### Human renal biopsy samples.

Renal biopsies had been performed as part of routine clinical diagnostic investigation. Twenty patients with acute renal tubular necrosis and 34 patients with no detectable lesions verified by renal biopsy were enrolled in this study from the Department of Nephrology of Xinqiao Hospital. The exclusion criteria included inflammatory and autoimmune-associated disease, hypohepatia, polycystic kidney disease, diabetes, and pregnancy. BUN and Scr of all patients were assayed. Kidney biopsies were obtained from these patients for immunohistochemical staining.

### Generation of renal tubule–specific Rest-knockout mice.

*Rest^RTKO^* mice were obtained from Cyagen Biosciences. *Rest^fl/fl^* mice were crossed with *Cdh16-Cre* mice to generate tubule-specific *Rest*-knockout mice (*Cdh16-Cre* × *Rest^fl/fl^*, designated *Rest^RTKO^*). Male *Rest^fl/fl^* littermates with no Cre expression served as controls. All mice were in a C57BL/6J background. Mouse genotyping was performed using genomic DNA isolated from tails by PCR at 2 weeks of age (primers shown in Supplemental Table 2).

### Animals.

Male C57BL/6J mice (8 weeks old) were purchased from Chongqing Tengxin Bioscience. Male mice at around 8 weeks old were subjected to renal IRI or sham operation. They were housed 6 to 8 per cage under a 12-hour light/12-hour dark cycle at 25°C, with humidity at 40%–70%.

### Statistics.

Statistical analyses were performed with GraphPad Prism 8 using a 2-tailed, paired Student’s *t* test, 1-way analysis of variance (ANOVA), or Pearson’s correlation test. A *P* value of less than 0.05 was considered statistically significant. All data were obtained from at least 3 independent experiments.

### Study approval.

Patient tissue sample collection was approved by the Ethics Committee of Second Affiliated Hospital of Army Medical University (2018-006-01), and all the study participants provided written informed consent. All the animal experiments were approved by the Animal Ethics Committee of Second Affiliated Hospital of Army Medical University (AMUWEC20224530) and performed in accordance with the NIH *Guide for the Care and Use of Laboratory Animals* (National Academies Press, 2011).

### Data availability statement.

The data sets used and/or analyzed during the current study are available from the corresponding author on reasonable request. The RNA-seq files have been deposited in the Sequence Read Archive (SRA) database (https://www.ncbi.nlm.nih.gov/sra/) with accession number PRJNA859283.

## Author contributions

YH and JZ designed the study, supervised the experiments, and revised the manuscript. SG performed the experiments and drafted the manuscript. AZ, MY, WX, SQ, and YL carried out the in vitro experiments and data analysis. XG, JX, KY, LX, and TH took part in the in vivo studies and statistical analysis. All authors approved the final version of the manuscript.

## Supplementary Material

Supplemental data

Supporting data values

## Version 1 06/08/2023

Electronic publication
