# Supplementary material for: REST contributes to AKI-to-CKD transition through inducing ferroptosis in renal tubular epithelial cells
Source: JCI Insight. 2023 Jun 8;8(11):e166001. doi: 10.1172/jci.insight.166001 (PMC10393228; doi:10.1172/jci.insight.166001)
Supplement: Supplemental data [file jciinsight-8-166001-s077.pdf]

Supplemental Information

This PDF file includes:

1. Supplemental text
2. Supplemental Tables 1 to 9
3. Supplemental Figures 1 to 13
4. References

Supplemental text

1. Materials and Methods

Drug treatment. In vitro experiments of HK2 cells: the following reagents were used at indicated concentrations: Erastin (MedChemExpress, HY-15763, New Jersey, USA) , RSL3 (MedChemExpress, HY-100218A), Deferoxamine (MedChemExpress, HY-B0988), Ferrostatin-1 (Selleckchem, S7243, Houston, TX, USA), Z-VAD-FMK (Selleckchem, S7023), 3-Methyladenine (Selleckchem, S2767), Necrostatin-1 (Selleckchem, S8037). In HT 1080 cells, RSL3 (MedChemExpress, HY-100218A) was used to induce ferroptosis.

IRI and Cisplatin induced mouse models. Mice were randomly assigned to sham or IRI groups. n = 8 mice per group. After skin antisepsis, mice were anesthetized and kept on a homeothermic plate. After exposing both kidneys, the IRI-induced AKI was established in mice by inducing ischemia for 30 min by clamping the renal pedicle with nontraumatic bulldog clamps, followed by 24 h blood reperfusion. Ferrostatin-1 was

intraperitoneally injected to *Rest*^{RTKO} mice and *Rest*$^{fl/fl}$ mice on the onset of reperfusion to evaluate the additional benefits of ferrostatins and REST-deficiency in AKI(1, 2). For the IRI-induced AKI to CKD model, bilateral renal ischemia for 35 min was followed by reperfusion for 2 or 4 weeks. The same operations were conducted in sham groups except for induction of IRI.

To construct the cisplatin-induced AKI model, 30 mg/kg of cisplatin was intraperitoneally injected into mice and they were sacrificed 3 days later, as described previously (3). Blood samples and kidneys were collected at the indicated times. One kidney was fixed in 4% paraformaldehyde for histological analyses, while the other was snap-frozen for subsequent molecular analysis.

Renal function, histology, immunofluorescence and immunohistochemical staining. Serum creatinine and BUN were determined to monitor renal function. Creatinine was measured by using high-performance liquid chromatography (Waters, MA, USA). BUN was measured using a urea assay kit (Nanjing Jiancheng Bioengineering Institute, Nanjing, China). The paraffin-embedded kidney was cut into a thickness of 5 μm sections and was stained with hematoxylin-eosin (HE), Masson, or used for immunohistochemistry and immunofluorescence. Tubular injury was evaluated in a blinded manner and random tissue sections were assessed based on the percentage of damaged tubules: 0, no damage; 1, < 25%; 2, 25–50%; 3, 50–75%; 4, > 75% (4). Results for each item were added to yield the ATN score, and images were obtained with a Digital slice scanner (Kfbio Technology of Health, Ningbo, China).

Immunofluorescence staining were observed by Zeiss 880 (Carl Zeiss AG, Germany). The intensities of the images were detected by Image J software (National Institutes of Health, USA).

The antibodies against 4HNE (ab48506), Fibronectin (ab2413) and α-SMA (ab5694) were bought from Abcam Biotechnology (Cambridge, MA, USA). The antibody against REST (2590-R) was obtained from Biosis (Beijing, China). LTL was bought from vector Laboratories Inc (San Francisco, California, USA).

Cell culture and treatment. Human proximal tubular epithelial cells (HK2 cells), rat renal tubular epithelial cells (NRK52E cells) and Human fibrosarcoma cells (HT1080 cells) were bought from the American Tissue Culture Collection (ATCC, Manassas, VA, USA). HK2 cells were cultured in F12 medium containing 10% fetal bovine serum (Mediatech Inc., Herndon, VA, USA). NRK52E cells and HT1080 cells were cultured in DMEM medium containing 10% fetal bovine serum (Mediatech).

HR model of HK2, primary renal tubular epithelial cells of mice and NRK52E cells was induced according to 24h hypoxia (94% N_2 + 1% O_2 + 5%CO_2, glucose and serum-free DMEM/F12 medium) followed by 1h, 3h, 6h, 9h, 12h of reoxygenation (95% air and 5% CO_2, DMEM/F12 medium with 10% FBS.

For cisplatin injury model, cells were incubated with cisplatin for 24 h.

Mouse primary tubule isolation and cell culture. Tubular epithelial cells (TECs) were isolated from 4-week-old WT or *Rest*$^{fl/fl}$ male mice kidneys as previous described (5) (6). Kidneys were collected immediately after the mice were sacrificed. Renal cortexes

were minced into pieces (~ 1 mm^3) and washed by 0.1 ml ice-cold Dulbecco's phosphate buffered saline (DPBS). Fragments were digested with 0.1% Collagenase II for 8 minutes. After digestion, DMEM/F12 medium (containing 10% of fetal bovine serum (FBS)) were added to stop the Collagenase activity. Then the tissue homogenate was sieved through 70 μm nylon mesh (BD Falcon, Franklin Lakes, NJ, USA) and centrifuged. Cells were centrifuged at 1000 rpm for 3 minutes. These renal tubules were collected after an ultracentrifugation density gradient. Cells were washed with DPBS and was resuspended in DMEM/F12 medium with 10% fetal bovine serum (Corning, NY, USA). To obtain *REST*$^{-/-}$ primary tubule epithelial cells, Ad-Cre adenovirus (HANBio, AP21031807, Shanghai, China) were used to infect cells for 72h. Infection efficiency was estimated under fluorescence microscope by the presence of GFP-positive cells (data not shown).

Overexpression or downregulation of target genes. For interfere *REST* or *GCLM*, HK2 cells or primary renal tubular epithelial cells of mice were transfected with control siRNA or siRNA against *REST* or *GCLM* by using Lipofectamine 3000 (Invitrogen, Carlsbad, CA, USA), according to the manufacturer's protocol. The siRNA sequences are described in Supplemental Table 3. Control or *REST* overexpression plasmids were transfected using Lipofectamine 3000 (Invitrogen, L3000075) in Opti-MEM (Hyclone, Logan, UT, USA), according to the manufacturer's protocol. Then, cells were treated with HR and were harvested for the subsequent experiments.

RNA extraction and quantitative real-time PCR. Total RNA was extracted from HK2 cells, NRK52E cells, primary renal tubular epithelial cells of the mouse kidney tissues by using an RNA Purified Total RNA Extraction Kit (Beyotime Biotechnology, R0077S, Shanghai, China) according to the manufacturer's instructions and reversely transcribed into cDNA using an RT Master Mix for qPCR kit (MedChemExpress, HY-K0511A). Real-time PCR was performed with a SYBR Green qPCR kit (MedChemExpress, HY-K0501). Primers are described in Supplemental Table 4, 5 and 6. Each experiment was performed at least three times.

Generation of inducible GPX4 knockdown cellular model for ferroptosis. The knockdown of *GPX4* in HK2 cells (iGPX4KD) was obtained by lentiviral infection using Tet-pLKO-puro vector with a cloned sequence of shRNA specific for *GPX4* (RabbitBio, Chengdu, China) (7). The shRNA sequences are described in Supplemental Table 7. The obtained pool of cells was selected with 2 μM Puromycin (Beyotime Biotechnology, ST551). Next, cells were seeded at 1 cell/well in a 96-well plate in the presence of the selecting antibiotic. Growing clones were screened for ferroptotic cell death induction upon doxycycline (Dox) incubation (Selleckchem, S5159) and the most potent clone was selected for further experiments. For these experiments, iGPX4KD cells were induced for 48 hours in the presence of 1 μM Dox (Selleckchem, S5159). 1 μM fer-1 was co-incubated with Dox for a positive control. siRNAs against REST were transfected 48 hours before Dox induction.

Western Blot. Briefly, protein samples were boiled for 15 min, electrophoresed in 8-12% SDS polyacrylamide gel, and transferred onto PVDF membranes (Merck Millipore, Billerica, MA, USA). The blots were blocked with Quick block solution (Beyotime Biotechnology, P0252) for 30 minutes at 37 °C. Then the corresponding primary and secondary antibodies were incubated to visualize the protein. The signal was detected by an enhanced chemiluminescence reagent (ProteinSimple, Santa Clara Valley, CA, USA). The densitometry of protein bands was quantified through Image J software (National Institutes of Health, USA).

The antibody against GCLM (ab126704), DHFR (ab288373), Lamin B1 (ab16048), Fibronectin (ab2413), GSDMD (ab209845) and pro-caspase1+p10+p12 (ab179515) were bought from Abcam (Cambridge, MA, UK). The antibodies against GPX4 (381958), β-actin (T200068-8F10), and ACSL4 (R24265) were obtained from Zenbio(Chengdu, China). The antibody against REST (07-579) was obtained from Merck Millipore. The antibodies against GCLC (12601-1-AP), α-SMA (14395-1-AP) were obtained from proteintech (Wuhan, China) and the antibody against FSP1 (sc-377120) was obtained from Santa Cruz Biotechnology (Dallas, TX, USA). LC3 (12741), P62 (5114), Bcl-2 (3498) and Bax (2772) were obtained from Cell Signaling Technology (CST, Danvers, MA, USA).

LDH, MDA, GSH and GCL activity levels assay. The relative LDH, MDA, GSH and GCL activity concentration in kidney tissues and cells was assessed with LDH (Solarbio, BC0685, Beijing, China) or MDA (Beyotime Biotechnology, S0131S) or GSH

(Nanjing Jiancheng Bioengineering Institute, A006-1-1), or GCL activity assay kit (Sangon, D799622) according to the manufacturer's instructions. Individual contents of MDA, GSH, and GCL activity were measured at 532, 412 and 660 nm. Total protein concentration was measured using the BCA method (Beyotime Biotechnology, P0009) at 532 nm respectively with a microplate reader.

Cell viability assay. HK2 cells were seeded into 96-well plates at 3000 cells per well. After treatment, the cell viability was measured by using the cell counting kit-8 (CCK-8) assay (MedChemExpress, HY-K0301). 100 μl of fresh medium containing 10 μl of CCK-8 solution was added to the cells, followed by incubation for 2 h at 37 °C. The absorbance at 450 nm was measured at indicated times.

ROS and Lipid ROS assay using flow cytometer. After exposed to HR injury, cells were washed with serum-free DMEM and incubated with DCFH-DA (Beyotime Biotechnology, S0033M) or BODIPY-C11 dye (Invitrogen, D3861) at 37 °C for 20 min. Both ROS and lipid ROS were analyzed using Accuri C6 flow cytometry (BD Biosciences, Franklin Lake, NJ, USA). The data were calculated by using the FlowJo Software.

PI/Calcein-AM staining. HK2 cells or HT1080 cells were washed with PBS and incubated with PI/Calcein-AM(Meilunbio, Dalian, China) at 37 °C for 30min after they were exposed to HR injury or treated with drugs. Then cells were observed by Zeiss 880 (Carl Zeiss AG, Germany).

Construction of reporter plasmids and point mutation. Putative REST binding sites in the *GCLM* promoter region are listed in Supplemental Table 8. Various lengths of the human *GCLM* promoter region were amplified by PCR using the genomic DNA of HK2 cells as a template. The fragments including *GCLM-2000* (−2000 to +200), *GCLM-1700* (−1700 to +200), *GCLM-1000* (−1000 to +200), *GCLM-500* (−500 to +200), *GCLM-200* (−200 to +200), and *GCLM-100* (−100 to +200) were separately cloned into a pGL3-basic vector (Promega, Madison, WI, USA) after digestion with HindIII, and the recombinant reporter plasmids were separately named as pGL3-*GCLM*-P6, pGL3-*GCLM*-P5, pGL3-*GCLM*-P4, pGL3-*GCLM*-P3, pGL3-*GCLM*-P2, and pGL3-*GCLM*-P1. The pGL3-*GCLM*-M2, containing point mutations in the REST binding element (GCCGCAAGCAAAGAGCCAATC, the mutated bases are underlined), was generated with MutanBEST kit (Takara) using pGL3-*GCLM*-P2 (−200 to +200) as a template. Negative control mutation plasmid pGL3-*GCLM*-M3, containing point mutations in the *GCLM* binding element (CGAGCGGCAGAGGGAGTCC, the mutated bases are underlined), was also generated, using pGL3-*GCLM*-P2 (−500 to +200) as a template.

Luciferase reporter constructs and dual-luciferase reporter assay. The recombinant reporter plasmids were co-transfected with pcDNA3.1 vector (Promega, Madison, Wisconsin, USA) or *REST* overexpression plasmids and Renilla plasmids into renal tubular epithelial cells using Lipofectamine 3000. Luciferase activity was detected

using The Dual-Luciferase Reporter Assay System (Promega, E1910). Firefly luciferase activity was normalized against Renilla activity.

ChIP and ChIP-qPCR. ChIP assays were performed by using a Simple Enzymatic ChIP Kit (Invitrogen, No. 26157) according to the manufacturer's instructions. After treatment, cells were fixed by 1% formaldehyde for 10 min. Next, cells were lysed in sodium dodecyl sulfate lysis buffer. The resulting chromatin was sonicated to shear DNA to an average length between 200 to 1000 bp, and immunoprecipitated with 3 μg of primary antibody against REST overnight. Non-specific IgG was used as a technical control. The precipitated DNA was amplified by PCR and qPCR with primers (−203 to −43) that covered the REST binding sites (−152 to −132). Total DNA (Input) served as a positive control. The immunoprecipitated DNA was analyzed by qPCR, for which the specific primers are listed in Supplemental Table 9.

RNA-Seq Profiling. Total RNA was extracted from HK2 cells using a TaKaRa RNAiso Plus reagent. Library construction and RNA sequencing were conducted by Majorbio. Briefly, cells were divided into three groups: (1) control, (2) HR control, and (3) HR +*siREST*, each with four repeats. A total amount of 1 μg RNA per sample was used as input material for RNA sample preparations. Sequencing libraries were generated using a TruSeq™ RNA sample preparation Kit (Illumina Inc., San Diego, CA, USA) according to the manufacturer's recommendations, and index codes were added to attribute sequences to each sample. mRNA was isolated according to the polyA selection method by oligo(dT) beads and then fragmented by fragmentation buffer.

Next, double-stranded cDNA was synthesized using a SuperScript double-stranded cDNA synthesis kit (Invitrogen) with random hexamer primers (Illumina). Subsequently, the synthesized cDNA was subjected to end-repair, phosphorylation, and 'A' base addition according to Illumina's library construction protocol. After quantified by TBS380, the paired-end RNA-seq sequencing library was sequenced with an Illumina HiSeq xten/NovaSeq 6000 sequencer (2 × 150 bp read length).

Gene Ontology (GO) enrichment analysis of differentially expressed genes and KEGG pathways were implemented by the cluster profile R package, in which gene length bias was corrected. Transcripts expression with |Log2 Fc| > 1.5, and P values < 0.05 were deemed statistically significant.

Mitochondrial morphology observation by transmission electron microscopy. Briefly, 1 mm^3 fresh renal cortex or primary renal tubular epithelial cells or HK2 cells aggregate was quickly placed in an electron microscopy fixative at 4 °C. Tissues were embedded and cut into 60–80 nm ultrathin sections and then subjected to uranium lead double staining. Transmission electron microscopy was used to observe the mitochondria and image acquisition (8).

Bioinformatics analysis. The binding sites of REST in *GCLM* promoter region were predicted by using the JASPAR database (http:/ /jaspar.genereg.net) (9).

2. Supplemental Tables

Supplemental Table 1. Clinical data of ATN and non-ATN patients examined.

Control subjects

Number	Age(year)	Sex	Scr (mg/dL)	BUN (mg/dL)
1	35	M	0.8937	16.576
2	18	M	0.5984	18.004
3	53	F	0.7783	14.224
4	22	F	0.6912	11.676
5	50	M	0.6980	10.248
6	43	F	0.9830	13.972
7	46	M	0.7998	16.436
8	25	F	0.8948	16.240
9	17	M	0.8903	14.588
10	50	F	0.9921	15.344
11	53	M	0.8360	7.448
12	37	F	0.6787	11.312
13	48	M	0.9661	10.024
14	44	M	0.8688	16.016
15	49	F	0.9434	9.912
16	49	F	0.9570	13.552
17	54	F	0.8009	11.004
18	49	F	0.8088	12.964
19	30	M	0.7613	18.480
20	54	M	0.6618	8.036
21	33	M	0.5803	10.808
22	50	M	0.7081	13.244
23	46	F	0.8111	12.964
24	25	F	0.6821	13.608
25	37	M	0.8812	13.804
26	51	F	0.9740	10.612
27	19	F	0.6210	12.992
28	31	M	0.5339	10.612
29	19	F	0.6041	12.880
30	60	M	0.8699	8.512
31	50	M	0.7443	9.436
32	50	F	0.6052	14.028
33	47	F	0.6844	7.980
34	57	F	0.6810	10.864

Subjects with acute tubular necrosis

Number	Age(year)	Sex	Scr (mg/dL)	BUN (mg/dL)
1	42	F	1.0769	21.504
2	54	F	1.4016	40.908
3	52	F	3.9265	35.896
4	42	F	1.3710	17.444
5	50	M	1.4163	16.240
6	53	F	1.2161	11.032
7	60	F	2.7738	64.064
8	25	M	2.5860	43.932
9	35	M	2.4864	30.268
10	40	M	2.8462	35.672
11	59	M	2.6109	22.988
12	55	M	2.4921	58.016
13	33	F	1.8812	31.192
14	47	F	1.9921	30.016
15	28	M	2.1821	37.156
16	51	M	1.0645	11.452
17	46	F	1.1991	11.788
18	51	F	1.5803	16.352
19	63	M	5.4581	40.740
20	46	F	4.2353	53.816

Supplemental Table 2. Primers for tail PCR genotyping.

Gene	Primers	Product length
Rest$^{fl/fl}$	F1: 5’-AGAGACGCTCAACTATCACCTTC-3’	218 bp (*Rest-flox*)
	R1: 5’-GACAAGAGAACACTTGCACCCATA-3’	150 bp (WT)
Cdh16-	F2: 5’- GCAGATCTGGCTCTCCAAAG -3’	420 bp
Cre	R2: 5’- AGGCAAATTTTGGTGTACGG -3’	

Supplemental Table 3. The sequence sets for siRNA.

Primer name		Primer Sequence (5'-3')	
human	*REST-si-1*	Sense (5'-3')	GCAAGAGCUCGAAGACCAAdTdT
		Antisense (5'-3')	UUGGUCUUCGAGCUCUUGCdTdT
	REST-si-2	Sense (5'-3')	GAGCGAGUCUACAAGUGUAdTdT
		Antisense (5'-3')	UACACUUGUAGACUCGCUCdTdT
	REST-si-3	Sense (5'-3')	AGUUCACAGUGCUAAGAAAdTdT
		Antisense (5'-3')	UUUCUUAGCACUGUGAACUdTdT
mouse	*Gclm-si-1*	Sense (5'-3')	GAAGAUAAAUCCCGAUGAAdTdT
		Antisense (5'-3')	UUCAUCGGGAUUUAUCUUCdTdT
	Gclm-si-2	Sense (5'-3')	GAUUGAAGAUGGAGUUAAUdTdT
		Antisense (5'-3')	AUUAACUCCAUCUUCAAUCdTdT
	Gclm-si-3	Sense (5'-3')	GCACCUCUGAUCUAGACAAdTdT
		Antisense (5'-3')	UUGUCUAGAUCAGAGGUGCdTdT
	NC	Sense (5'-3')	UUCUCCGAACGUGUCACGUdTdT
		Antisense (5'-3')	ACGUGACACGUUCGGAGAAdTdT

Supplemental Table 4. The primer sets for human.

Gene (human)	Primers	Product length
REST	Forward: 5'-CGGTTGGGGATAACAACTTTTCA-3'<br>Reverse: 5'-TCTACGACGCTGAGT TCCAAA-3'	127bp
β-actin	Forward: 5'-CATGTACGTTGCTATCCAGGC-3'<br>Reverse: 5'-CTCCTTAATGTCACGCACGAT-3'	250bp
FSP1	Forward: 5'-ACAGCCAGCCCTTCCTC-3'<br>Reverse: 5'-TGCCCTCACAGACAGACAC-3'	143bp
GSS	Forward: 5'-ATGAACCCTGCCCCACT-3'<br>Reverse: 5'-AGGCTATGCCCCTCCAC-3'	113bp
GCLC	Forward: 5'-AACACAGACCCAACCCAGAG-3'<br>Reverse: 5'-CCGCATCTTCTGGAAATGTT-3'	201bp
GCLM	Forward: 5'-GCCACCAGATTTGACTGCCTTT-3'<br>Reverse: 5'-CAGGGATGCTTTCTTGAAGAGCTT-3'	119bp
DHFR	Forward: 5'-CAGCGAGCAGGTTCTCAT-3'<br>Reverse: 5'-ACTATGTTCCGCCCACAC-3'	136bp
GPX4	Forward: 5'-TTGCCGCCTACTGAAGC-3'<br>Reverse: 5'-ATGTGCCCGTCGATGTC-3'	141bp
MMP24	Forward: 5'-CCAGGGCGTCTGAAGTG-3'<br>Reverse: 5'-GAGCGAGGTCAGCAAGG-3'	125bp
HMOX1	Forward: 5'-CTCCTCTCGAGCGTCCTCAG-3'<br>Reverse: 5'-AAATCCTGGGGCATGCTGTC-3'	107bp
RELL2	Forward: 5'-CTCATGTCGGAACCACAGCCT-3'<br>Reverse: 5'-AGGCCTCAGCATTGGCTTCA-3'	268bp
SEZ6L2	Forward: 5'- GGAGAGAGATCGGGGTGAGT-3'<br>Reverse: 5'- ATGAAGCAGTTCAGCCAGGG-3'	192bp
KCNK3	Forward: 5'- TCGTGTGCACCTTCACCTAC-3'<br>Reverse: 5'- CCCTGGCTGAGGTTGTAGC-3'	136bp
SNAP25	Forward: 5'- ACCAGTTGGCTGATGAGTCG-3'<br>Reverse: 5'- ACACGATCGAGTTGTTCTCCT-3'	124bp
RTN2	Forward: 5'-TTTGTCCCACCAGATCACC-3'<br>Reverse: 5'-GAAGATGGCACCCACGA-3'	132bp
SPINK1	Forward: 5'-GTGCGGTGCAGTTTTCA-3'<br>Reverse: 5'-TTCCATCAGTCCCACAGAC-3'	199bp
TRIM16	Forward: 5'-GAGCCTATGGGGTTCAGAT-3'<br>Reverse: 5'-CAGCCAGATGCGATGAC-3'	132bp

Supplemental Table 5. The primer sets for mouse.

Gene (mouse)	Primers	Product length
β-actin	Forward: 5'-TGTTACCAACTGGGACGACA-3'<br>Reverse: 5'-GGGGTGTTGAAGGTCTCAAA-3'	165bp
Kim1	Forward: 5'-ACATATCGTGGAATCACAACGAC-3'<br>Reverse: 5'-ACTGCTCTTCTGATAGGTGACA-3'	114bp
Ngal	Forward: 5'-GCAGGTGGTACGTTGTGGG-3'<br>Reverse: 5'-CTCTTGTAGCTCATAGATGGTGC-3'	95bp
Rest	Forward: 5'-CGGGTGAAGCCAACCAAAAG-3'<br>Reverse: 5'-CTCTAACAGGCACCAAGCCA-3'	124bp
FSP1	Forward: 5'-GCCTTGCCCTTCTCACA-3'<br>Reverse: 5'-GCTGGATCTGCTTCACCA-3'	127bp
Gss	Forward: 5'-CTCCGACGTGGTGACGTA-3'<br>Reverse: 5'-GGGTACTGGTGAGGGGAAA-3'	52bp
Gpx4	Forward: 5'-TTCTCAGCCAAGGACATCG-3'<br>Reverse: 5'-CACTCAGCATATCGGGCAT-3'	149bp
Gclm	Forward: 5'-GCCTTACAAGAAGCATCCC-3'<br>Reverse: 5'-GCCCTCAAA ACTCAACACA-3'	65bp
Gclc	Forward: 5'-GCACGTTGCTCATCTCTTT-3'<br>Reverse: 5'-TCAGACTCGTTGGCATCA-3'	81bp
Dhfr	Forward: 5'-CTCAGGGCTGCGATTTC-3'<br>Reverse: 5'-ACGATGCAGTTCAATGGTC-3'	104bp
Hmox1	Forward: 5'-CCTCACAGATGGCGTCACTT-3'<br>Reverse: 5'-TGGGGGCCAGTATTGCATTT-3'	200bp
Rell2	Forward: 5'-GCAACGCGAGTAGGCATT-3'<br>Reverse: 5'-AGGATGTGGGCCGGTAG-3'	120bp
Mmp24	Forward: 5'-CTGTCCCTCTGCCTCCTG-3'<br>Reverse: 5'-GGGCTGCTCATACCCACT-3'	115bp
Sez6l2	Forward: 5'-CCTTTCCCCTCCCACTTT-3'<br>Reverse: 5'-AGGTTATCCCCTTGTTCGG-3'	77bp
Trim16	Forward: 5'-CCGGTCTATGCTGCTTTC-3'<br>Reverse: 5'-CCTCTCCCAGATCCACAA-3'	67bp
Snap25	Forward: 5'-ACGAAGCACCACTGACTTG-3'<br>Reverse: 5'-CCCTCCTCTGCATCTCCT-3'	106bp
Rtn2	Forward: 5'-CCACACCCTCTCCCACTC-3'<br>Reverse: 5'-GGGCAGCGTCCTGACTAT-3'	150bp
Spink1	Forward: 5'-TTCTTCTCAGTGCTTTGGC-3'<br>Reverse: 5'-ATTCCGTCAGTCCCACAC-3'	139bp
Kcnk3	Forward: 5'- CTACCTGCAACCCAGTGGAG-3'<br>Reverse: 5'- CCATAGCCCCACACATACCC-3'	90bp

Supplemental Table 6. The primer sets for rat.

Gene (rat)	Primers	Product length
β-actin	Forward: 5'-CGCGAGTACAACCTTCTTGC-3'<br>Reverse: 5'-CGCAGCGATATCGTCATCCA-3'	81bp
Rest	Forward: 5'-CGGACGCCGGTAGCGA-3'<br>Reverse: 5'-ATAATGAGCTGAGGTGCCGC-3'	168bp

Supplemental Table 7. The sequence sets for shRNA.

Primer name		Primer Sequence (5'-3')
GPX4-sh-1	Sense (5'-3')	CCGGAAGGACATCGACGGGCACATGCTCG<br>AGCATGTGCCCGTCG ATGTCCTTTTTTG
	Antisense (5'-3')	AATTCAAAAAAAGGACATCGACGGGCAC<br>ATGCTCGAGCATGTGCCCGTCGATGTCCTT
GPX4-sh-2	Sense (5'-3')	CCGGAAGAGATCAAAGAGTTCGCCGCTCG<br>AGCGGCGAACTCTTTGATCTCTTTTTTG
	Antisense (5'-3')	AATTCAAAAAAAGAGATCAAAGAGTTCGC<br>CGCTCGAGCGGCGAACTCTTTGATCTCTT
GPX4-sh-3	Sense (5'-3')	CCGGAAGCGCTACGGACCCATGGAGCTCG<br>AGCTCCATGGGTCCGTAGCGCTTTTTTG
	Antisense (5'-3')	AATTCAAAAAAAGCGCTACGGACCCATGG<br>AGCTCGAGCTCCATGGGTCCGTAGCGCTT

Supplemental Table 8. Putative binding sequences of REST in *GCLM* promoter region.

**Name**	**Start**	**End**	**Strand**	**Predicted sequence**
REST	76	96	-	TACACTACCACTGACAGTGTA
REST	370	390	-	GTTCATGTCCTTTGTAGGGAA
REST	1750	1768	-	TGACCGCCAGGGGGAGCCC
REST	1849	1869	-	GCCGCAGGCCAAGGGCCAGTC

Supplemental Table 9. The primer sets for ChIP.

Gene	Primers	Product length
GCLM	Forward: 5'-GCCACGCTCTCTCGACC-3'<br>Reverse: 5'-AGCCGAGAAAGTGCTTCGTA-3'	161bp

3. Supplemental Figures

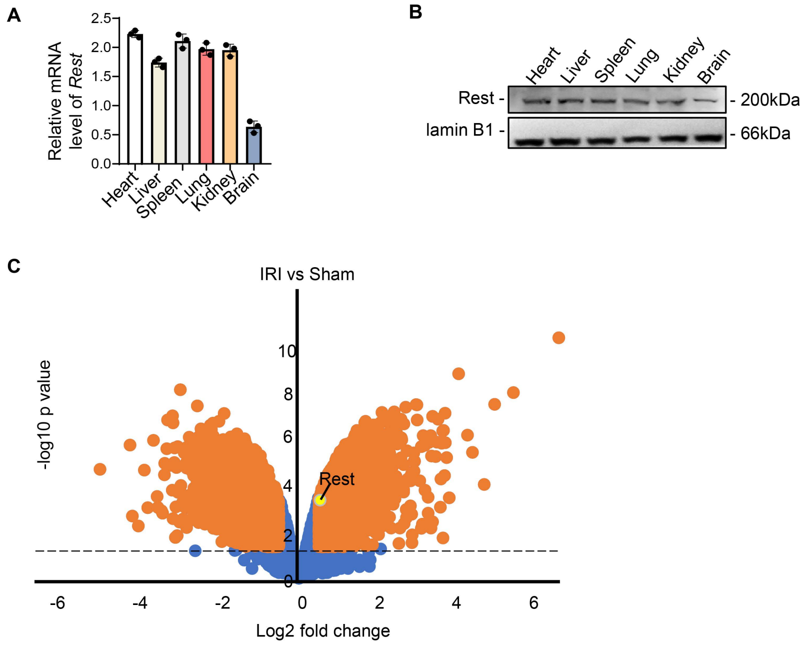

Supplemental Figure 1. REST Expression in major organs and the renal tubules from sham and AKI mice. (**A** and **B**) qPCR (**A**) and Western blot (**B**)analysis of REST expression levels in different organs. (**C**)Volcano plot of sham and IRI-induced AKI (GSE52004).

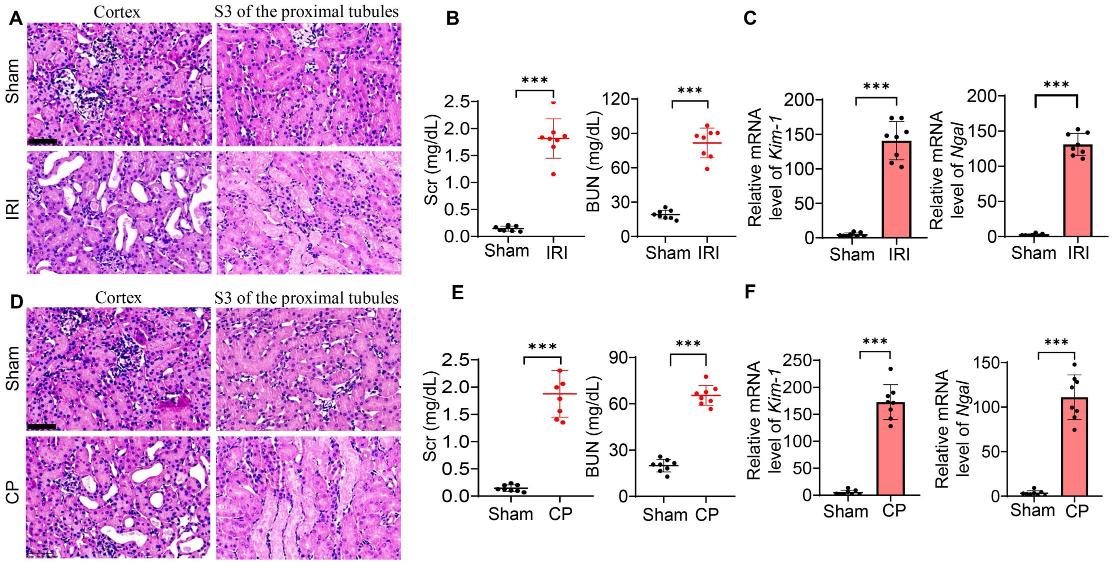

Supplemental Figure 2. Establishment of IRI-induced AKI and cisplatin-induced AKI. (**A**-**F**) Representative HE staining (**A** and **D**), serum levels of Scr and BUN (**B** and **E**) and the levels of *Kim-1* and *Ngal* (**C** and **F**) in IRI-induced and cisplatin-induced AKI. Scale bar: 50 μm, (n = 8 mice per group). Data shown are mean ± SD and analyzed by two-tailed unpaired Student's t-test (**B**, **C**, **E** and **F**). *** $P < 0.001$.

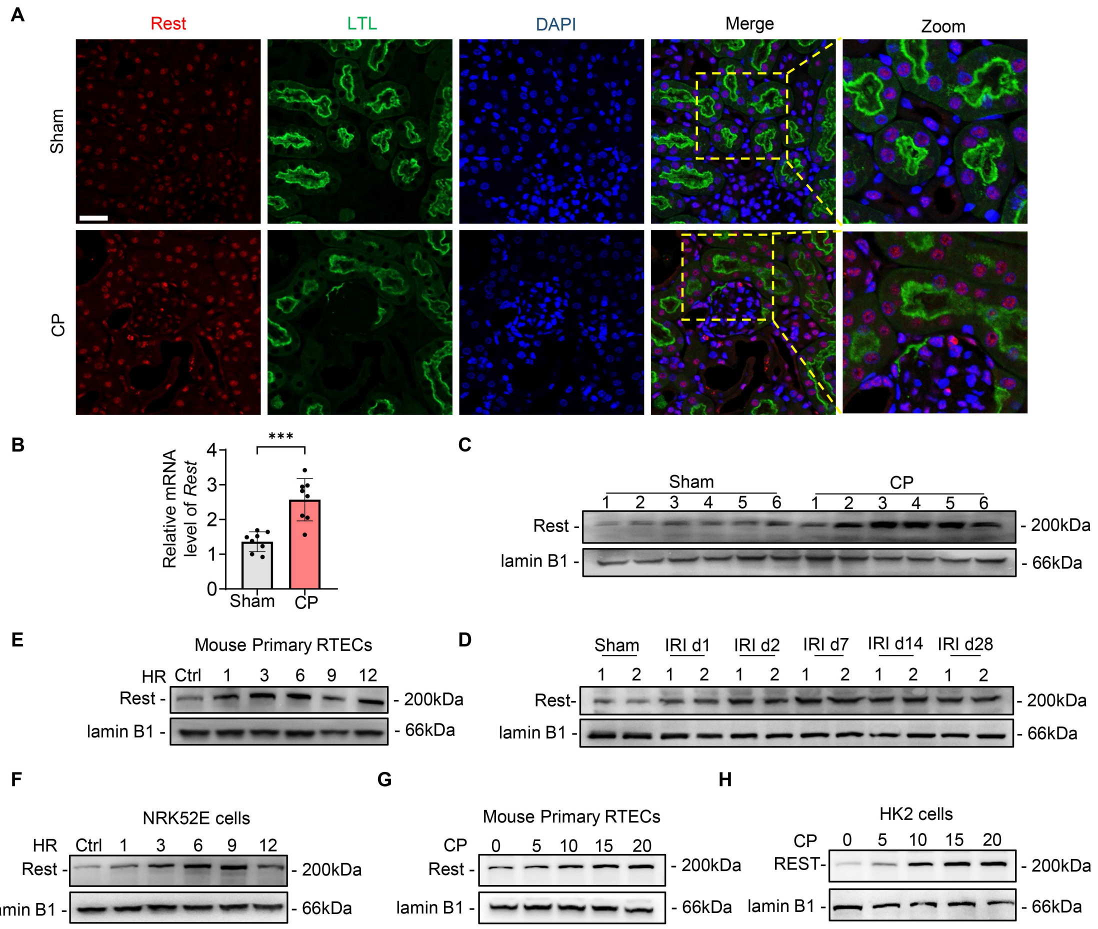

Supplemental Figure 3. Rest is upregulated in renal tubules from mice with AKI and AKI-to-CKD. (**A-C**) immunofluorescence staining (**A**), qPCR (**B**) and Western blot analysis (**C**) of Rest in cisplatin-induced AKI group. Scale bar: 50 μm, (n = 8 mice per group). (**D**) Protein level of Rest after IRI 1, 3, 7, 14 and 28 days in mice. (**E** and **F**) Western blot analysis of Rest in primary renal tubular epithelial cells of mouse (mouse primary RTECs) (**E**) and NRK52E cells (**F**) after 24 hours of hypoxia and subsequent different reoxygenation duration time. (**G** and **H**) Western blot of REST in HK2 (**G**) and mouse primary RTECs (**H**) treated with a concentration gradient of cisplatin. Data were shown as mean ± SD and analyzed by two-tailed unpaired Student's t-test (**B**). *** $P < 0.001$.

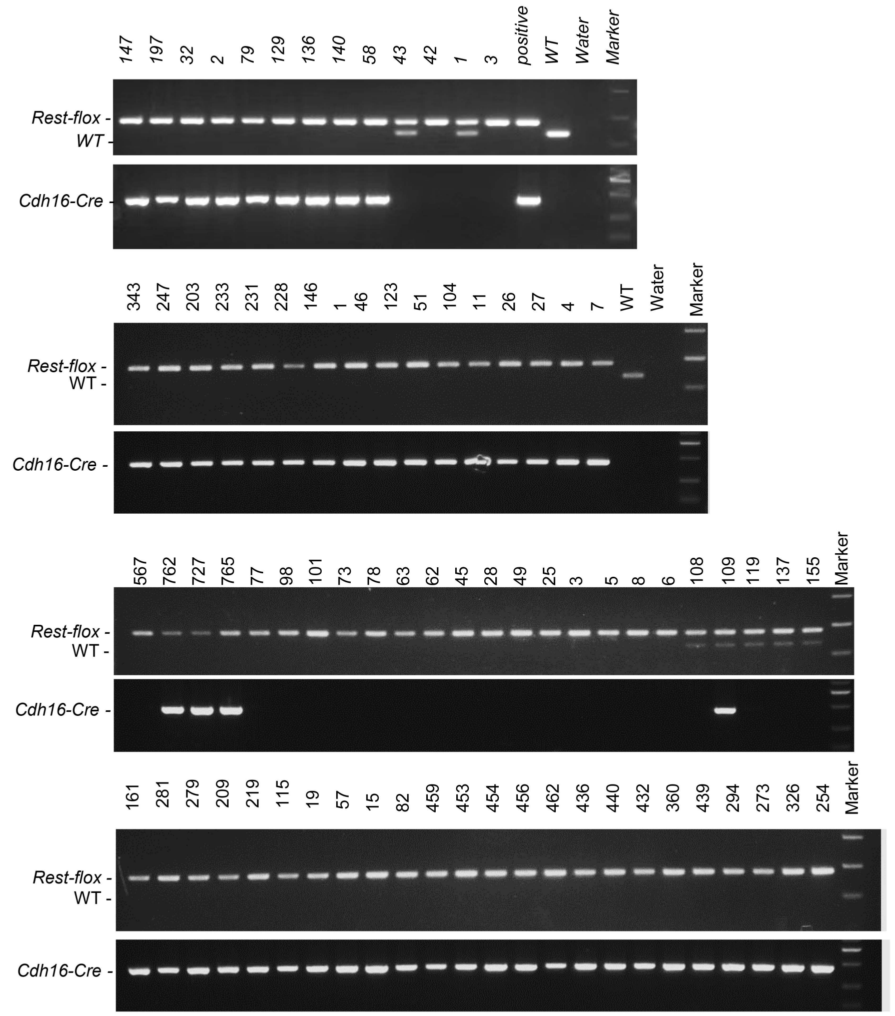

Supplemental Figure 4. Genetic identification of *Rest* conditional knockout mice.

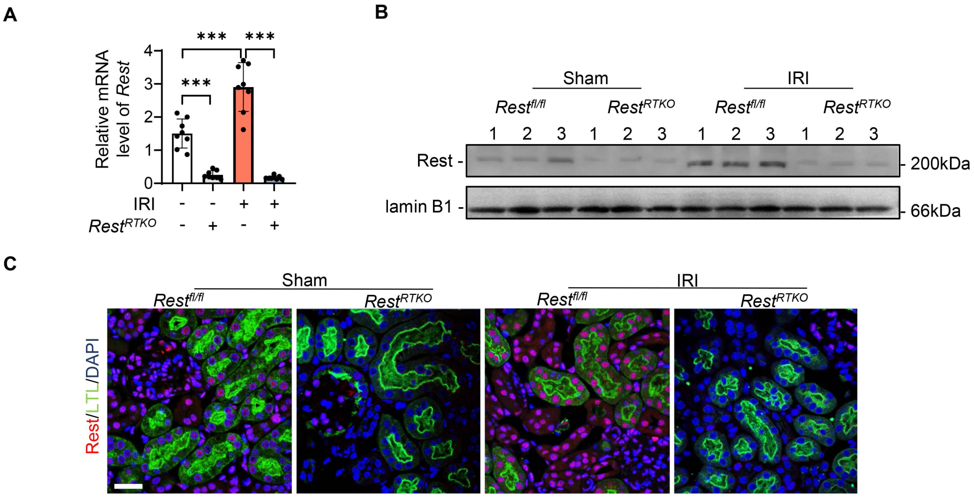

Supplemental Figure 5. Identification of *Rest* conditional knockout mice. (A-C) The qPCR(**A**), Western blot analyses (**B**) and immunofluorescent analyses (**C**) of Rest in *Rest*$^{fl/fl}$ and *Rest*^{RTKO} mice with or without IRI. Scale bar: 50 µm. (n = 8 mice per group). Data shown are mean ± SD and analyzed by one-way ANOVA. *** $P <$ 0.001.

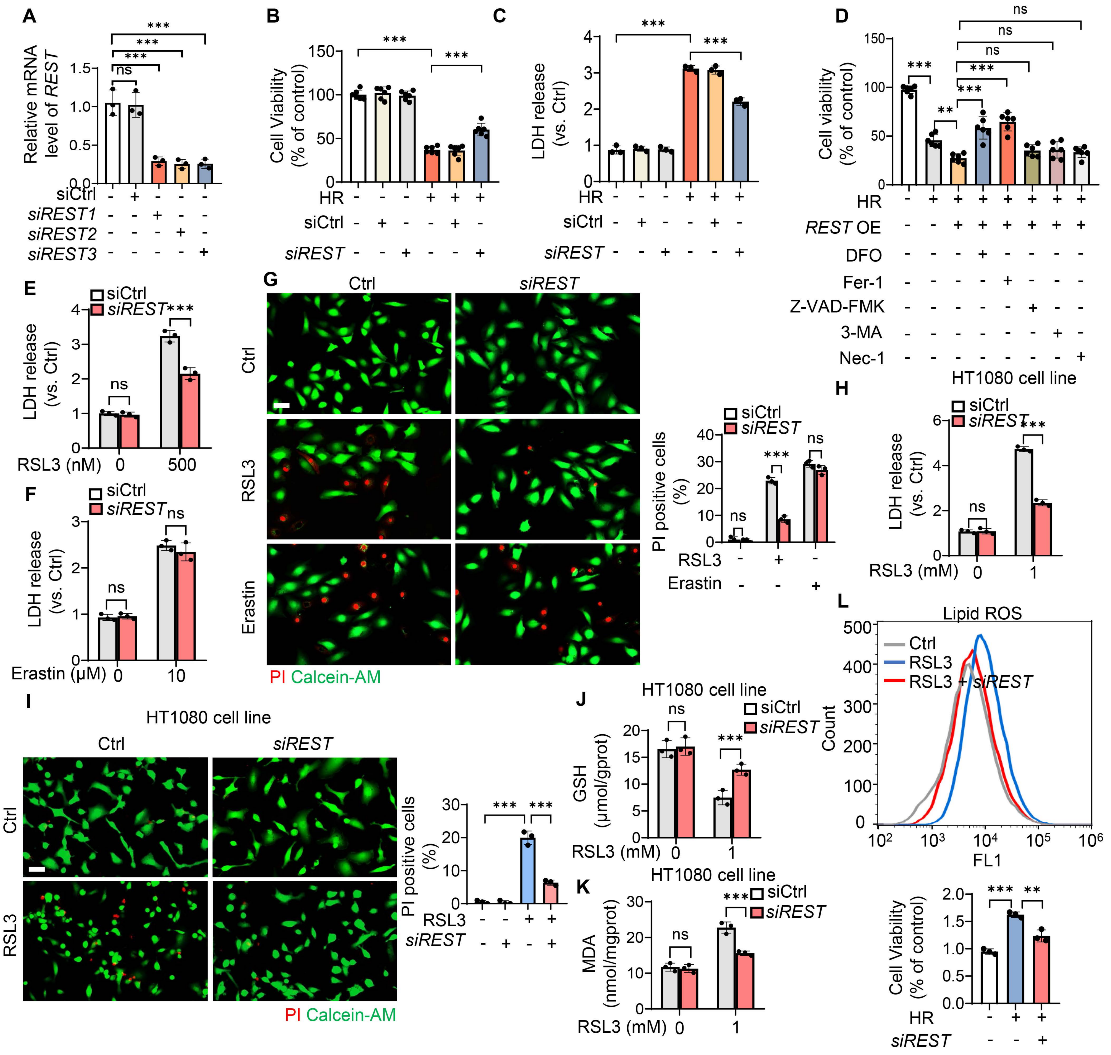

Supplemental Figure 6. ***REST* knockdown improves cell viability and inhibits ferroptosis.** (**A**) mRNA of *REST* after conducted with control or siRNAs against *REST* (*siREST*). (n = 3). (**B** and **C**) Cell viability (**B**) and LDH release (**C**) of HK2 cells under normoxia or HR injury transfected with control or *siREST*. (n = 3). (**D**) HK2 cells were transfected with REST overexpression plasmids and incubated with deferoxamine (DFO) (100 μM), ferrostain-1(fer-1) (1 μM), Z-VAD (10 μM), 3-Methyladenine (3-MA) (3 mM) or necrostatin-1(nec-1) (10 μM) respectively under HR condition, and then cell viability was detected. (n = 3). (**E-G**) HK2 cells were transfected with *siREST* or control and incubated with RSL3 (0.5 μM) or Erastin (10 μM). Then LDH release and PI/Calcein-AM staining were detected. (n = 3). (**H-L**) HT1080 cells were transfected with control or si*REST*, and then cultured normally or incubated with RSL3(1 μM) for detection of LDH (**H**), PI/Calcein-AM (**I**), GSH (**J**), MDA (**K**), and lipid ROS (**L**). (n = 3). Data shown are mean ± SD and analyzed by two-tailed unpaired Student's t-test (**E-H**, **J** and **K**) and one-way ANOVA (**A-D**, **I** and **L**). * $P < 0.05$, ** $P < 0.01$, *** $P < 0.001$, ns: no significance.

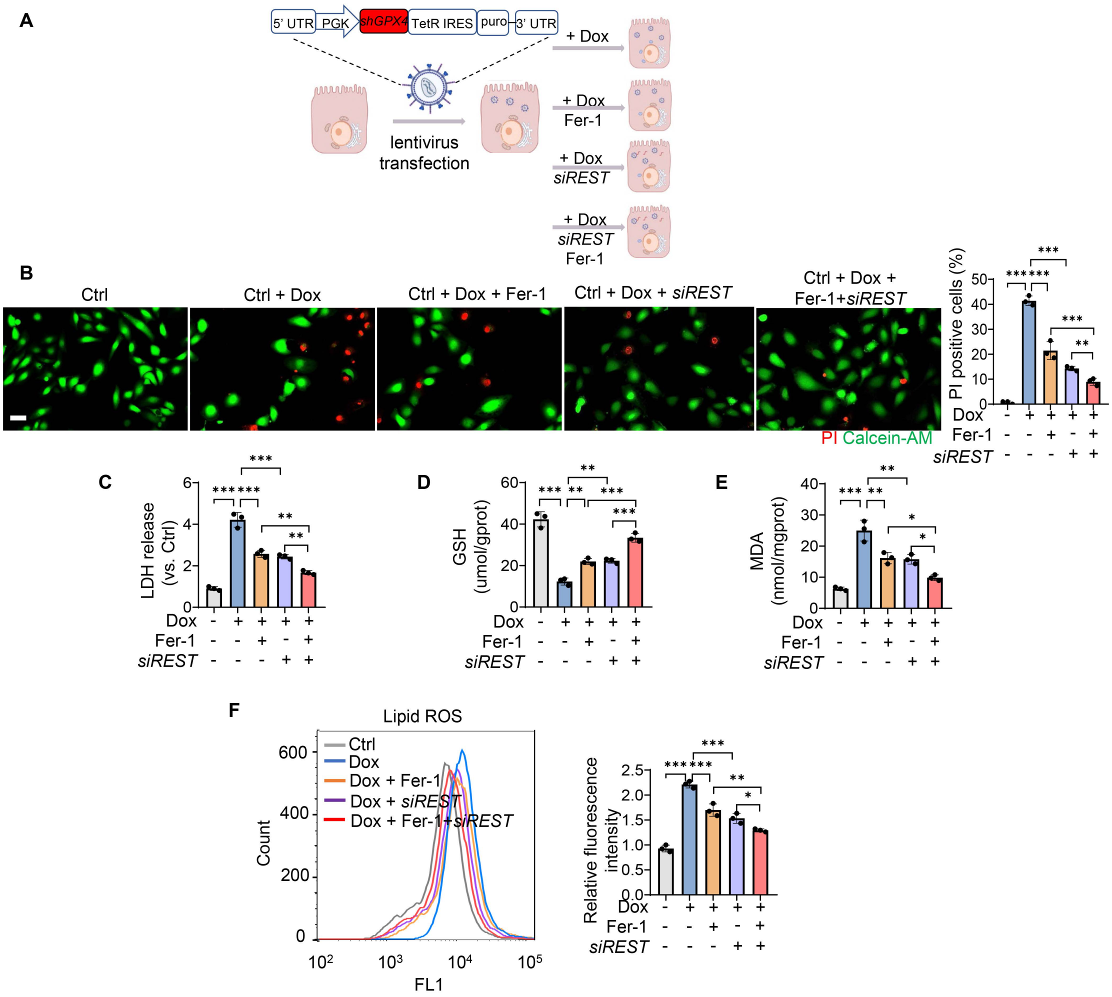

Supplemental Figure 7. Knockdown of *REST* can overcome ferroptosis induced by *GPX4*-deficiency. (**A**) HK2 cells were infected with lentivirus which can knockdown *GPX4* by doxycycline (Dox) treatment. Then they were divided into five groups: (1) Ctrl group was cultured normally, (2) Dox group was incubated with Dox, (3) Dox+Fer-1 group was co-incubated with Dox and fer-1, (4) Dox+*siREST* group was transfected with siRNAs against REST for 48 h and then added with Dox, (5) Dox+Fer-1+*siREST* group was transfected with siRNAs against REST for 48 h and then added with Dox and Fer-1. (**B-F**) these cells were used to observe PI/Calcein-AM staining (**B**), LDH release(**C**), GSH production (**D**), MDA levels (**E**) and lipid ROS accumulation (**F**). Data shown are mean ± SD and they were analyzed by one-way ANOVA (**B-F**). (n = 3). * $P < 0.05$, ** $P < 0.01$, *** $P < 0.001$.

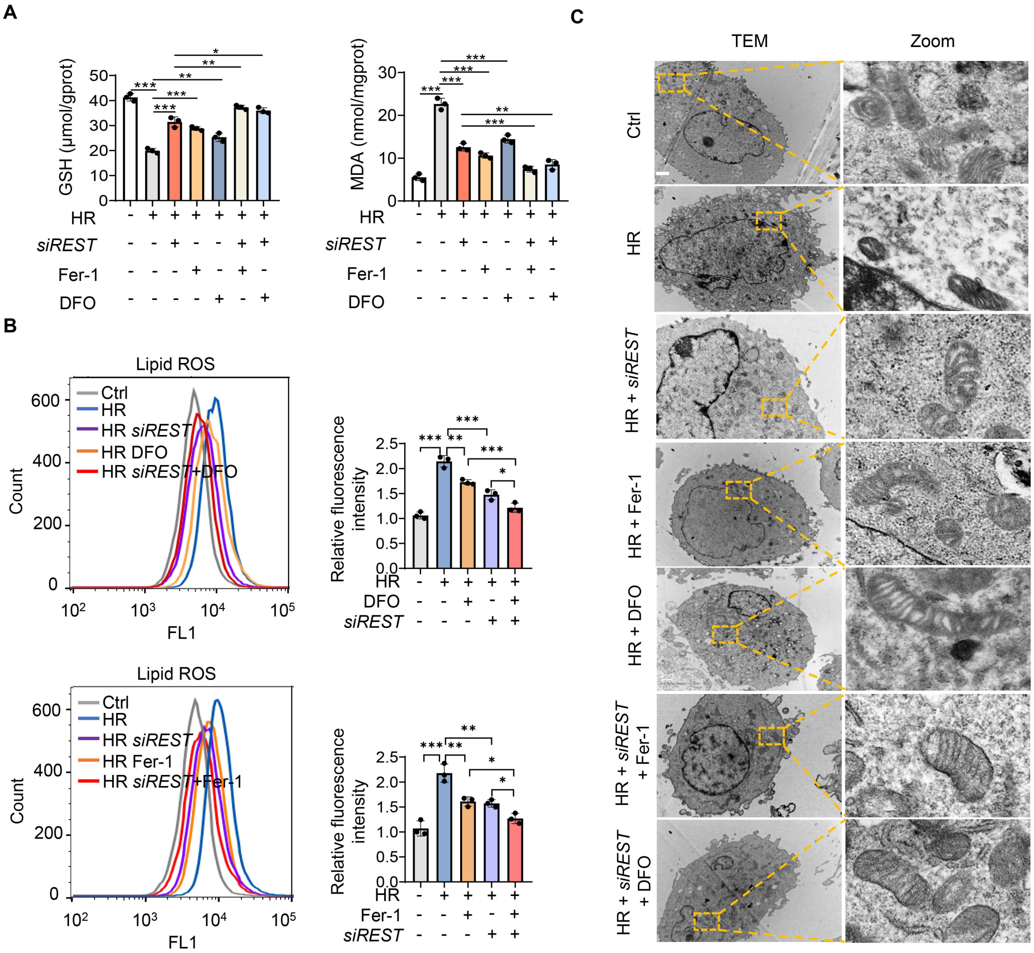

Supplemental Figure 8. REST-deficiency has additional benefits on deferoxamine and ferrostatin-1 to attenuate ferroptosis. HK2 cells were incubated with DFO or Fer-1 separately with control or *siREST* transfection, and then exposed to HR injury. Then they were used to detect GSH and MDA levels (**A**), lipid ROS accumulation (**B**), and TEM observations (**C**). (n = 3). Data shown are mean ± SD and analyzed by one-way ANOVA (**A** and **B**). * $P < 0.05$, ** $P < 0.01$, *** $P < 0.001$, ns: no significance.

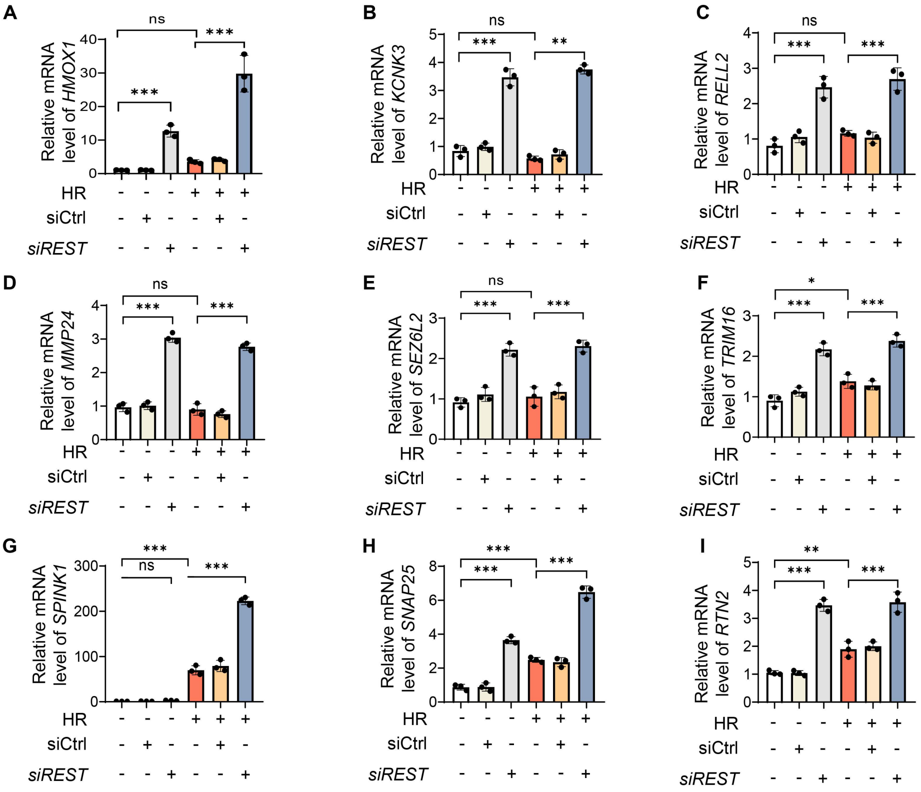

Supplemental Figure 9. Screen of top ten upregulated genes in HK2 cells with *REST* knockdown. qPCR of the top ten upregulated genes except *GCLM* in RNA-seq after *REST* was knockdown in HK2 cells under HR condition (**A-I**). (n = 3). Data shown are mean ± SD and analyzed by one-way ANOVA (A-I). * $P < 0.05$, ** $P < 0.01$, *** $P < 0.001$, ns: no significance.

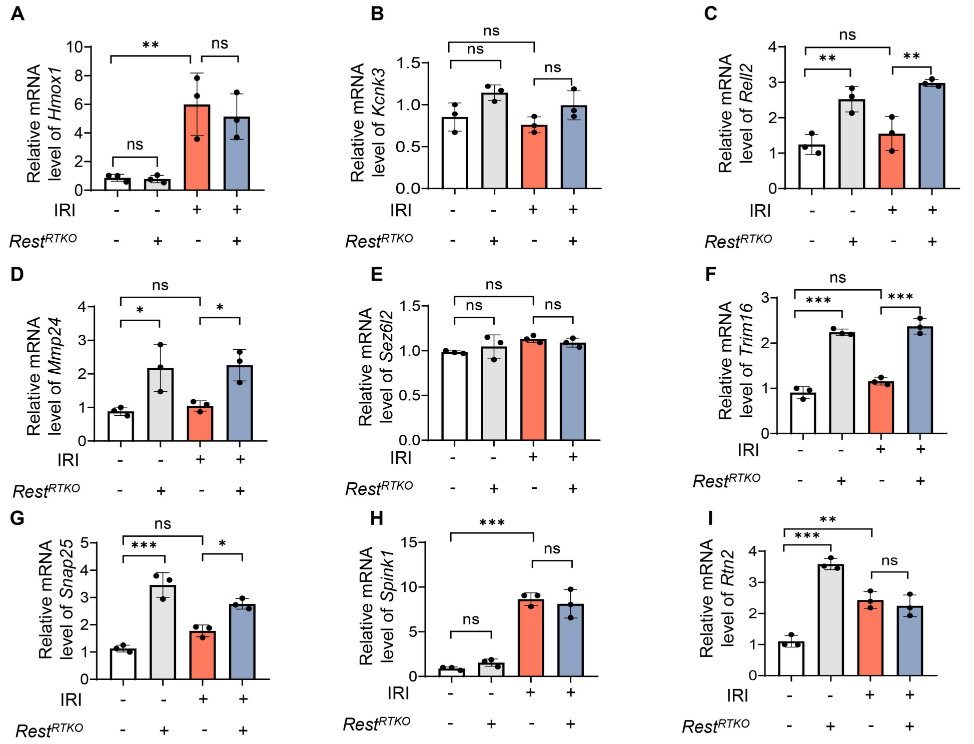

Supplemental Figure 10. Screen of top ten upregulated genes in *Rest*^{RTKO} mice. qPCR of the top ten upregulated genes except *GCLM* in RNA-seq from renal tubules in *Rest*^{RTKO} mice of IRI-induced AKI (**A-I**). Data shown are mean ± SD and analyzed by one-way ANOVA (**A-I**). (n = 3). * $P < 0.05$, ** $P < 0.01$, *** $P < 0.001$, ns: no significance.

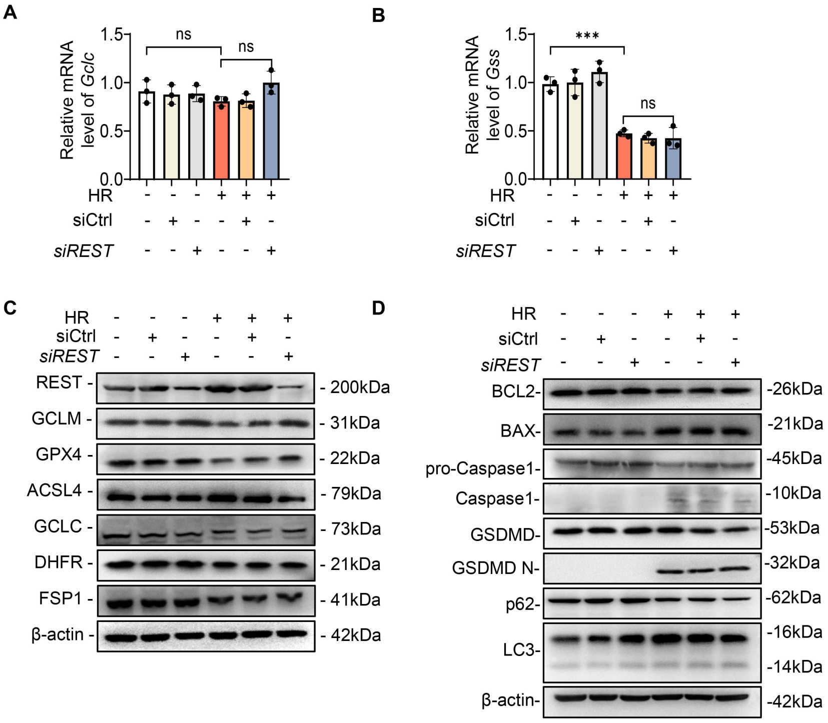

Supplemental Figure 11. Knockdown of *REST* has no effect on other ferroptosis-related genes or other major forms of cell death. HK2 cells were transfected with control or *siREST*, and then exposed to HR injury. (**A** and **B**) Represented relative mRNA levels of *GCLC* (**A**) and *GSS* (**B**) between different groups. (n = 3). (**C**) Western blot analyses of the protein levels of REST, GCLM, GPX4, ACSL4, GCLC, DHFR and FSP1 in different groups. (**D**) Western blot analyses of the protein levels of Bcl-2, Bax, Caspase-1, GSDMD, LC3 and P62 in different groups. Data shown are mean ± SD and they were analyzed by one-way ANOVA (**A** and **B**). *** $P < 0.001$, ns: no significance.

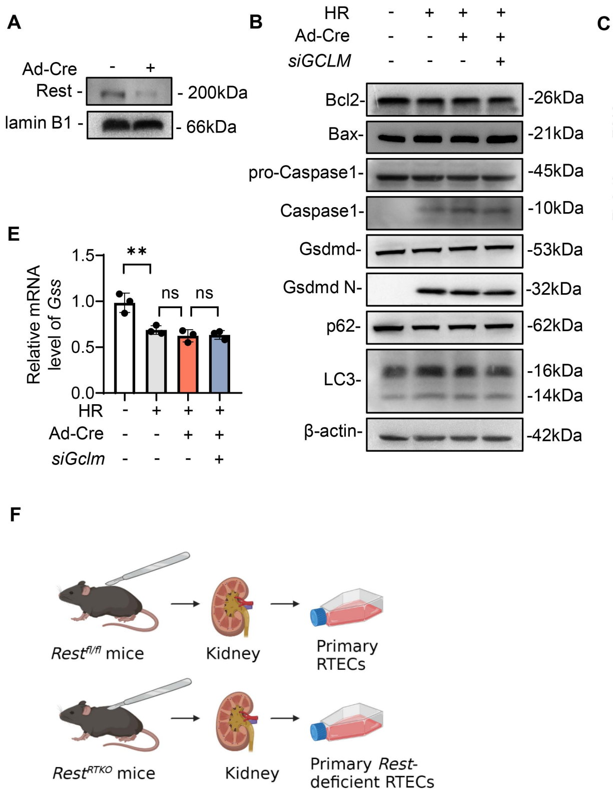

Supplemental Figure 12. Knockdown of *Gclm* has no effect on other major forms of cell death in REST-deficiency cells under HR condition. (**A**) The protein level of Rest after Ad-Cre Adenovirus infection in primary RTECs. (**B**-**E**) Primary RTECs from $Rest^{fl/fl}$ mice were infected with Ad-Cre Adenovirus and transfected with siRNAs against *GCLM,* and then exposed to HR injury. Cells were collected to detect: protein levels of Bcl2, Bax, Caspase-1, Gsdmd, LC3 and p62 (**B**), mRNA levels of *Gclm* (**C**), *Gclc* (**D**) and *Gss* (**E**). (n = 3). (**F**) Primary REST-deficient RTECs were isolated and cultured from $Rest^{RTKO}$ mice. Data shown are mean ± SD and they were analyzed by one-way ANOVA (**C-E**). ** $P < 0.01$, *** $P < 0.001$, ns: no significance.

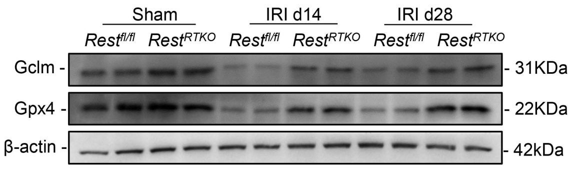

Supplemental Figure 13. Renal tubular epithelial cell-specific knockout of *Rest* restores the expressions of Gclm and Gpx4 in AKI-to-CKD mice. Western blot analysis of the expressions of Gclm and Gpx4 in the kidneys from *Rest*$^{fl/fl}$ and *Rest*^{RTKO} mice with or without IRI on reperfusion day 14 and day 28. (n = 8 mice per group).

4. References

1. Hu Z, Zhang H, Yi B, Yang S, Liu J, Hu J, et al. VDR activation attenuate cisplatin induced AKI by inhibiting ferroptosis. *Cell death & disease.* 2020;11(1):73.
2. Deng F, Sharma I, Dai Y, Yang M, and Kanwar YS. Myo-inositol oxygenase expression profile modulates pathogenic ferroptosis in the renal proximal tubule. *The Journal of clinical investigation.* 2019;129(11):5033-49.
3. Liu J, Livingston MJ, Dong G, Tang C, Su Y, Wu G, et al. Histone deacetylase inhibitors protect against cisplatin-induced acute kidney injury by activating autophagy in proximal tubular cells. *Cell death & disease.* 2018;9(3):322.
4. Tang TT, Wang B, Wu M, Li ZL, Feng Y, Cao JY, et al. Extracellular vesicle-encapsulated IL-10 as novel nanotherapeutics against ischemic AKI. *Science advances.* 2020;6(33):eaaz0748.
5. Xiong J, Ran L, Zhu Y, Wang Y, Wang S, Wang Y, et al. DUSP2-mediated inhibition of tubular epithelial cell pyroptosis confers nephroprotection in acute kidney injury. *Theranostics.* 2022;12(11):5069-85.
6. Chung KW, Dhillon P, Huang S, Sheng X, Shrestha R, Qiu C, et al. Mitochondrial Damage and Activation of the STING Pathway Lead to Renal Inflammation and Fibrosis. *Cell Metab.* 2019;30(4):784-99 e5.

7. Wiernicki B, Maschalidi S, Pinney J, Adjemian S, Vanden Berghe T, Ravichandran KS, et al. Cancer cells dying from ferroptosis impede dendritic cell-mediated anti-tumor immunity. *Nature communications.* 2022;13(1):3676.

8. Huang Y, Wang S, Zhou J, Liu Y, Du C, Yang K, et al. IRF1-mediated downregulation of PGC1α contributes to cardiorenal syndrome type 4. *Nature communications.* 2020;11(1):4664.

9. Huang Y, Zhou J, Wang S, Xiong J, Chen Y, Liu Y, et al. Indoxyl sulfate induces intestinal barrier injury through IRF1-DRP1 axis-mediated mitophagy impairment. *Theranostics.* 2020;10(16):7384-400.
